# Soft computing models to predict the compressive strength of GGBS/FA- geopolymer concrete

**DOI:** 10.1371/journal.pone.0265846

**Published:** 2022-05-25

**Authors:** Hemn U. Ahmed, Azad A. Mohammed, Ahmed Mohammed

**Affiliations:** Civil Engineering Department, College of Engineering, University of Sulaimani, Kurdistan, Iraq; RMIT University, AUSTRALIA

## Abstract

A variety of ashes used as the binder in geopolymer concrete such as fly ash (FA), ground granulated blast furnace slag (GGBS), rice husk ash (RHA), metakaolin (MK), palm oil fuel ash (POFA), and so on, among of them the FA was commonly used to produce geopolymer concrete. However, one of the drawbacks of using FA as a main binder in geopolymer concrete is that it needs heat curing to cure the concrete specimens, which lead to restriction of using geopolymer concrete in site projects; therefore, GGBS was used as a replacement for FA with different percentages to tackle this problem. In this study, Artificial Neural Network (ANN), M5P-Tree (M5P), Linear Regression (LR), and Multi-logistic regression (MLR) models were used to develop the predictive models for predicting the compressive strength of blended ground granulated blast furnace slag and fly ash based-geopolymer concrete (GGBS/FA-GPC). A comprehensive dataset consists of 220 samples collected in several academic research studies and analyzed to develop the models. In the modeling process, for the first time, eleven effective variable parameters on the compressive strength of the GGBS/FA-GPC, including the Activated alkaline solution to binder ratio (l/b), FA content, SiO_2_/Al_2_O_3_ (*Si/Al*) of FA, GGBS content, SiO_2_/CaO (*Si/Ca*) of GGBS, fine (*F*) and coarse (*C*) aggregate content, sodium hydroxide (*SH*) content, sodium silicate (*SS*) content, (*SS/SH*) and molarity (*M*) were considered as the modeling input parameters. Various statistical assessments such as Root Mean Squared Error (RMSE), Mean Absolute Error (MAE), Scatter Index (SI), OBJ value, and the Coefficient of determination (R^2^) were used to evaluate the efficiency of the developed models. The results indicated that the ANN model better predicted the compressive strength of GGBS/FA-GPC mixtures compared to the other models. Moreover, the sensitivity analysis demonstrated that the alkaline liquid to binder ratio, fly ash content, molarity, and sodium silicate content are the most affecting parameter for estimating the compressive strength of the GGBS/FA-GPC.

## 1. Introduction

It is widely known that the production of Portland cement needs a considerable amount of energy and at the same time contributes to generating a huge volume of the total carbon dioxide (around 7%) to the atmosphere, directly and indirectly, the heating of limestone releases CO_2_ directly which is called calcination (50%), while the burning of fossil fuels to heat the kiln indirectly results in CO_2_ emissions, this is around 40 percent of cement emissions and finally around 10% for quarrying and transporting [1, 2]. Also, approximately 2.8 tons of raw materials are needed to manufacture one ton of cement; this is a resource-exhausting process that consumes many natural resources such as limestone and shale to produce clinkers cement [3]. Furthermore, approximately one trillion liters of mixing water are required to be used in the concrete industry annually [4]. In the same context, after the steel and aluminum industry, cement is one of the most energy-exhaustive construction materials that used around 110–120 kWh to produce one ton of cement in a typical cement plant alone [5]. Nevertheless, the majority of the cementing materials for concrete production are Portland cement (PC). Therefore, to decrease the environmental impact of PC, many researchers have been carried out to develop a new material to be an alternative to the PC [6]; among them, geopolymer technology was developed first by Davidovits in France, 1970 [7]. The green gas emission of geopolymer concrete (GC) is around 70% lower than the PC concrete due to the high consumption of waste materials in the mixed proportions of the GC [8, 9]. The applicability of the water film thickness (WFT) model to the rheological properties of SCPB was briefly assessed.

Additionally, an improved dynamic yield stress model considering the water content within flocs was proposed for contributing to an in-depth understanding of the rheological behavior of SCPB or CPB. It is found that despite the significant reduction in dynamic, static yield stress and equivalent plastic viscosity of SCPB with SP incorporation, there is a Critical Micelle Concentration that makes the characteristic time of the destructuration exhibit first an increasing and then decrease trend. Under steady-state shearing (100 s−1), the shear stress can be divided into three stages with time regardless of SP dosage. An exponential relationship is observed between the rheological parameters of SCPB and WFT. The proposed model can achieve high prediction accuracy and applicability. Moreover, the model’s effective solid concentration and packing density can be considered the important parameters of high integration [10, 11].

Geopolymers are one of the parts of mineral alumino-silicate polymers that generated from alkaline activation of different source materials that are rich in aluminosilicate materials, such as natural source materials like metakaolin, by-product industrial source materials like fly ash (FA), and the by-product of agro source materials such as rice husk ash (RHA) [12]. The microstructure of geopolymer materials is amorphous, and their chemical constituents are similar to the natural zeolitic materials. The mineral composition of the ash-based geopolymer and alkaline activators are the factors that affect the final product of the polymerization process. Also, the high temperature has usually accelerated the polymerization process [13, 14]. So it can be concluded that geopolymer is the third generation of cementing materials after lime and cement [15]. The mixed proportions of the geopolymer concrete are consist of aluminosilicate binder, fine and coarse aggregates, alkaline solutions, and water; the polymerization between these ingredients produces a solid concrete almost like normal concrete [16]. Binder source materials of the geopolymer concrete are those rich in alumino-silicates such as FA, GGBS, RHA, metakaolin, POFA, or any hybridization between these ashes with or without Portland cement. Of these, fly ash is the most commonly used as source material for making geopolymer concrete due to its low cost, abundance availability, and higher potential for preparation geopolymers [17]; also, FA has been used by researchers to replace Portland cement in different types of concrete and cementitious composites [18]. However, one of the drawbacks of using FA as a single source binder material for the production of the geopolymer concrete is required high temperature and oven or steam curing to active and accelerate the polymerization process to set and harden the concrete specimens, on the other hand, FA-based geopolymer concrete cured at the ambient condition has been showing low reactivity [19]. In addition, pure FA utilization for the preparation of geopolymer concrete leads to limited usage of this technology in precast construction [20] because most of the engineering applications are executed in the ambient environmental condition. Therefore, the researches have been carried out to tackle these problems, among of them using GGBS [21–24] as a partial replacement of FA to the production of geopolymer concrete since GGBS has a higher content of calcium oxide (CaO) as compared to FA which it is responsible for the strength gain of geopolymer concrete at ambient curing conditions. Moreover, strength improvement was reported in the literature with GGBS and FA-based geopolymer concrete [25, 26].

The polymerization mechanism could be briefly explained as follows; in the first stage, dissolution of the silicate and aluminum elements of the binder inside the high alkalinity aqueous solution produces ions of silicon and aluminum oxide. In the second stage, a mixture of silicate, aluminate, and aluminosilicate species, through a contemporary operation of poly-condensation-gelation further condensation, finally produces an amorphous gel [27]. Several factors could influence the performance of GC, such as type of binder, the concentration of the alkaline solution, the molarity of sodium hydroxide, sodium silicate to sodium hydroxide ratio, extra water, mix proportion, and curing method [28].

The compressive strength of all concrete composites, including GC, is one of the most remarkable mechanical properties. Usually, it gives a general performance about the quality of the concrete composites [29]. The compressive strength test is conducted by following the standard test methods of ASTM C39 [30] or BS EN 12390–3 [31]. In the literature, various studies have been conducted to investigate the influence of several mixture proportion parameters and curing conditions on the mechanical properties of GGBS/FA-GPC. For instance, Nath and Sarker [23, 32, 33] reported that the GGBS replacement level, content, and type of alkaline solution influence the fresh and hardened properties of GGBS/FA-GPC. One factor affecting the polymerization process is the type and quantity of the alkaline liquids by influencing the release of Si^4+^ and Al^3+^ from the base binders. Alkaline liquids of greater concentration are usually beneficial for increasing compressive strength up to an optimal range [34]. Furthermore, a variety of (Na_2_SiO_3_/ NaOH) ratios was used to prepare geopolymer concrete, for instance, a research study was carried out by Topark-Ngarm et al. [35], who used a different ratio of Na_2_SiO_3_/ NaOH, and they reported that with the increasing of Na_2_SiO_3_/ NaOH, compressive strength was increased.

Another important parameter that affects the performance of GGBS/FA-GPC is the curing condition of the samples. Generally, there are various types of curing regimes, namely, ambient curing [20, 36], heat curing [37, 38], and steam curing [39–41]. The polymerization process is rapidly increased with the increment of curing temperature, which makes the GC gain up to 70% of its final strength when the specimens are cured inside an oven at 65°C for 24 hr. beyond which there is a peripheral enhance in the compressive strength after 28 days of maturity [42, 43]. Further, heat curing regimes give higher compressive strength than the ambient curing condition for the same GC mixture [44–47].

Achieving an authoritative model for predicting the compressive strength of GC is essential regarding saving in time, energy, and cost-effectiveness and gives guidance about scheduling for the construction process and removal of framework elements [48]. The modeling of the compressive strength characteristic of the GGBS/FA-GPC is essential regarding the possibility of changing or validating the GC mix proportions [49]. By selecting appropriate mixing proportions, economical and efficient designs will be accomplished. Therefore, various researches have been tried to shorten the time of choosing a proper mix of proportions to get the targeted properties; among them is modeling with developing empirical equations. There are different ways to model construction materials’ characteristics, including statistical techniques, computational modeling, and nowadays developed techniques such as regression analysis [50, 51]. A variety of factors affect the compressive strength of the GGBS/FA-GPC; this leads to different compressive strength results; as a consequence, predicting compressive strength is a challenging task for researchers and engineers. Therefore, there is a need for numerical and mathematical models [52]. Due to the good ability of machine learning regarding prioritization, optimization, forecasting and planning were widely used in the various engineering fields [48]. In the literature, machine learning systems were used to model the various characteristics of different types of concrete composites such as compressive strength of green concrete [53], splitting tensile and flexural strength of recycled aggregate concrete [54], modulus of elasticity of recycled concrete aggregate [55, 56], the compressive strength of high volume fly ash concrete [57], the compressive strength of eco-friendly GC containing natural zeolite and silica fume [58], splitting tensile strength of fiber-reinforced concrete [59], compressive strength of self-compacting concrete modified with nano-silica [60], and so on.

There is a lack of measuring effects of several mixture proportion parameters and different curing regimes on the compressive strength of GGBS/FA-GPC at the 28 days in the literature. Also, according to the comprehensive and systematic review on the GGBS/FA-GPC, an authoritative and developed model that used various parameters to predict the compressive strength of GGBS/FA-GPC is very rare to be used by the construction industry. The majority of efforts have concerned a single-scale model without covering broad laboratory work data or various parameters. Moreover, more than one parameter affects the compressive strength of GGBS/FA-GPC. Therefore, in this study, for the first time, in a single developed model, influences of eleven parameters, such as alkaline solution/binder (*l/b*), fly ash (*FA*) content, SiO_2_/Al_2_O_3_ (*Si/Al*) of fly ash, ground granulated blast furnace slag (GGBS) content, SiO_2_/CaO of GGBS, fine aggregate (*F*) content, coarse aggregate (*C*) content, sodium hydroxide (*SH*) content, sodium silicate (*SS*) content, (*SS/SH*) ratio, and molarity (*M*) were investigated and quantified on the compressive strength of GGBS/FA-GPC by using different model techniques, namely Linear Regression (LR), Multi-logistic Regression (MLR), Artificial Neural Network (ANN), and M5P-Tree (M5P). They were used as predictive models for predicting the compressive strength of green GGBS/FA-GPC at 28 days by using 220 samples from the literature studies.

### 1.1 Research significance

Providing multiscale models to predict the compressive strength of GGBS/FA-GPC is the main scope of this study. Thus, a wide range of laboratory work data, about 220 tested specimens with various *l/b*, *FA*, *Si/Al*, *GGBS*, *Si/Ca*, *F*, *C*, *SH*, *SS*, *SS/SH*, and *M* were considered with different analysis approaches aiming: (i) to guarantee the construction industry to use the provided models without any theoretical; (ii) to carry out statistical analysis and recognize the influence of various parameters on the compressive strength of GGBS/FA-GPC; (iii) to quantify and provide a systematic multiscale model to predict the compressive strength of GGBS/FA-GPC with the mixture propositions containing a various range of parameters; (iv) to discover the most authoritative model to predict the compressive strength of GGBS/FA-GPC from different model techniques (LR, MLR, ANN, and M5P-Tree) using statistical assessment tools.

## 2. Materials and methods

**Fig 1:**
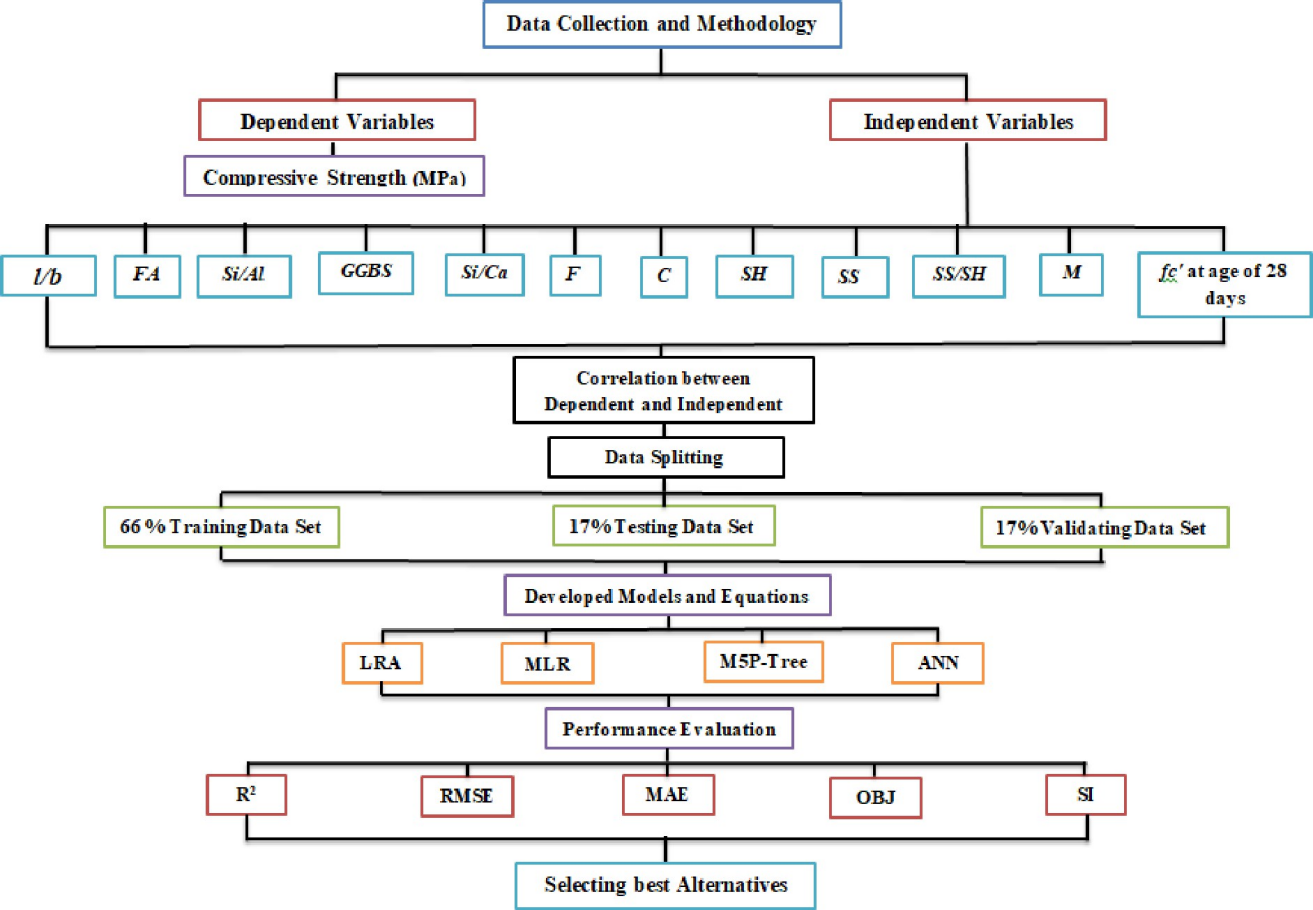
The flow chart diagram process followed in this study.

At the age of 28 days, 220 datasets were collected from previous GGBS/FA-GPC, and references are cited in this paper. There is a wealth of information on geopolymer concrete with various curing settings, specimen ages, and base source materials in the literature. On the other hand, the authors of this work utilize the measured compressive strength after 28 days of curing at room temperature. Those publications employed GGBS and fly ash (FA) as base source materials to manufacture the geopolymer concrete. The authors could incorporate additional datasets in the created models since the models required eleven input parameters. For example, if the mix proportions and any other model parameters of the investigation were not supplied, such studies were disregarded. The models were created using a bigger dataset collection, which contained 150 datasets. The second group consists of 35 datasets used to test the suggested models, and the third group consists of 35 datasets used to validate the supplied models [48, 60]. Table 1 shows the dataset ranges, including all significant parameters and the observed compressive strength of GGBS/FA-GPC at 28 days. The input dataset contains the following values: l/b ranges from 0.25 to 0.8, FA ranges from 0–639.4 kg/m3, Si/Al ranges from 1.41 to 7.77, GGBS ranges from 0–450 kg/m3, Si/Ca ranges from 0.66–2.04, F ranges from 459–756 kg/m^3^, C ranges from 915–1345 kg/m^3^, SH ranges from 34–122 kg/m^3^, SS ranges from A flow chart depicting the data gathering and modeling activity is shown in Fig 1.

**Table 1:** Summary of different GGBS/FA-GPC mixes.

Ref.	(*l/b*)	*FA* (kg/m^3^)	*(Si/Al)*	GGBS (kg/m^3^)	(Si/Ca)	*F* (kg/m^3^)	*C* (kg/m^3^)	*SH* (kg/m^3^)	*SS* (kg/m^3^)	(*SS/ SH*)	M	*fc′* (MPa)
[61]	0.4	300	2.07	150	0.97	742.7	1058	51.43	129	2.5	10–16	23–36
[62]	0.4–0.5	100–200	1.54	200–400	0.87	708–716	1050–1068	54–76	144–185	1.9–3.3	5.5–9	31–65
[63]	0.35–0.45	-	-	400–450	0.8	625–636	1154–1169	52–53	106–128	2.5	12–14	36–66
[64]	0.35	102–205	2.03	204–409	0.84	554	1293	41	102	2.5	10	46–60
[44]	0.38–0.46	350–450	2.16	-	-	540–575	1265–1343	38–52.5	95–131	2.5	16	13–14
[65]	0.45	266–380	2.36	38–114	1.01	660	1189	48.85	122	2.5	8	30–40
[66]	0.45	350–400	2.16	-	-	505–533	1178–1243	45–51	112–128	2.5	16	22–41
[67]	0.35	204–409	2.34	102–204	0.89	549	1290	41	102	2.5	10	24–54
[68]	0.4	394.3	2.31	-	-	646.8	1201.2	45.06	112.64	2.5	16	46.8
[22]	0.35–0.4	320–400	1.97	40–80	0.66	651–658	1209–1222	40–56	84–114	1.5–2.5	14	27–54
[69]	0.25–0.5	400–475	1.58	-	-	529–547	1235–1280	34–57	85–142	2.5	14	18–34
[70]	0.35	102–205	2.34	204–409	0.89	554	1293	41	102	2.5	10	53–58
[39]	0.35	408	1.65	-	-	647	1202	41	103	2.5	14	27–32
[71]	0.45	180	2.13	180	1	746	1120	46–64	81–115	1–2.5	6–14	8–18
[72]	0.6	390	1.61	-	-	585	1092	67	167	2.5	8–18	23–30
[73]	0.43	95–318	2.08	67–264	0.67	721–753	1111–1160	61–77	75–94	1.2	10	29–70
[74]	0.38	408	2.17	-	-	660	1201	41	103	2.5	10–16	25–33
[75]	0.4	274–437	1.41	43–206	1.22	740–767	915–948	69	171	2.5	8	28–71
[76]	0.45	500	2.4–2.9	-	-	575	1150	64.3	160.7	2.5	14	44–52
[77]	0.4–0.45	266–380	2.37	30–114	1.01	660	1178	45–69	103–131	1.5–2.5	8	31–43
[45]	0.4	350	0.48	-	-	650	1250	41	103	2.5	8	19
[78]	0.4–0.8	175–350	2.2	75–150	1.04	628–742	1007–1190	80	120	1.5	14	32–66
[23]	0.4	400	1.97	-	-	651	1209	45.7	114.3	2.5	14	26.5
[79]	0.36	106–382	2.11	42–318	1.01	577.54	1346	47	106	2.25	10	29–36
[46]	0.4	424.8	1.58	-	-	585.6	1183	63.36	95	1.5	14	32–46
[80]	0.35	168–420	1.95	168–420	0.98	617.23	1084	42	105	2.5	8	22–53
[81]	0.4	410	5.67	-	-	530.6	1044	67	117	1.74	10	10
[33]	0.35–0.4	340–400	1.97	40–60	0.66	651–655	1209–1218	40–46	100–114	2.5	14	25–47
[35]	0.5	414	2.27	-	-	588	1091	69–104	104–138	1–2	10–20	33–47
[82]	0.45	237	2.32	158	0.89	547	1277	52	129	2.5	8	28
[83]	0.4	394.3	1.95	-	-	554.4	1293	45	113	2.5	12	22
[84]	0.35–0.4	320–360	1.97	40–80	0.66	651–655	1209–1217	40–46	100–114	2.5	14	27–46
[85]	0.4–0.6	345–394	7.77	-	-	554	1294	45–83	94–148	1.5–2.5	8–16	7–22
[20]	0.35–0.4	300–400	2.02	40–100	0.98	637–671	1184–1246	46–53	93–114	1.5–2.5	10–12	21–56
[36]	0.4	394.3	1.8	-	-	554.4	1293	45	113	2.5	8	17.69
[86]	0.5	240–432	2.47	48–240	0.88	590	1090	69	171	2.5	10	27–70
[87]	0.4	350	1.82	-	-	483	1081	40	100	2.5	14	23.4
[88]	0.35	245–408	1.53	40–163	0.88	554	1294	41	103	2.5	8	16–45
[37]	0.65	639.4	2.67	-	-	639.4	959.4	121.8	304.5	2.5	8–12	20–32
[89]	0.4	197–355	3.54	39–197	2.04	554.4	1294	45	113	2.5	10	15–49
[90]	0.35	500	1.6	-	-	623	1016	70	105	1.5	14–16	22–27
[91]	0.4	350	2.13	-	-	645	1200	41	103	2.5	8	15–20
[92]	0.4–0.55	-	-	390–500	0.98	675–685	1031	60	150	2.5	12–16	42–55
[26]	0.45–0.6	359–523	2.2	-	-	459–525	1124–1298	108–118	108–118	1	10–15	15–37
[93]	0.3	-	-	415	1.02	784	1039	46	71	1.5	10	39.5
[32]	0.35–0.45	280–400	1.77	40–120	0.75	651	1209	40–64	96–114	1.5–2.5	14	26–56
[94]	0.6	192–347	2.43	38–192	0.95	602	1204	66.2	166	2.5	12	58–72
**Remarks**	**0.25**	**0**	**1.41**	**0**	**0.66**	**459**	**915**	**34**	**75**	**1**	**5.5**	**8**
	**-**	**-**	**-**	**-**	**-**	**-**	**-**	–	**-**	**-**	**-**	**-**
	**0.8**	**639.4**	**7.77**	**450**	**2.04**	**756**	**1345**	**122**	**305**	**3.3**	**20**	**72**

## 3. Statistical assessment

### a) Alkaline solution/binder (*l/b*)

**Fig 2:**
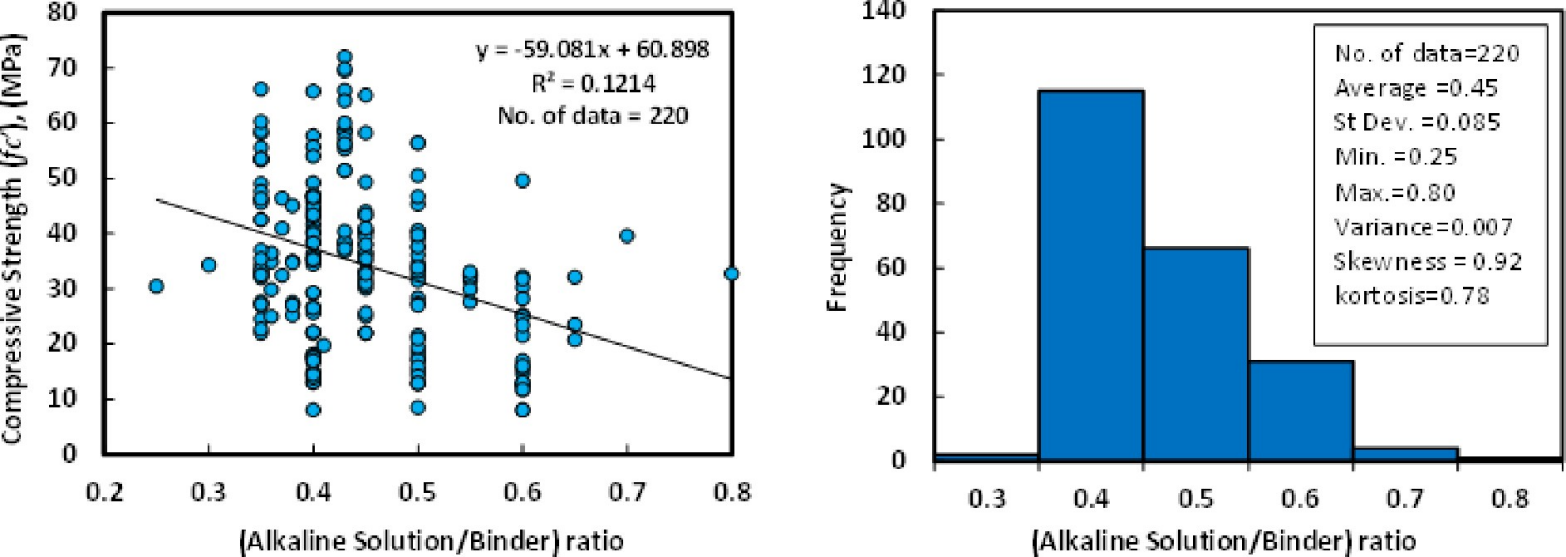
Relationship and a histogram of compression strength and alkaline solution/binder of GGBS/FA-GPC mixtures.

According to the dataset, which contains 220 data samples from past researches, the *l/b* ratio of the GGBS/FA-GPC was varied from 0.25 to 0.80 with an average variance, standard deviation, skewness, and kurtosis of 0.45, 0.007, 0.085, 0.92, and 0.78, respectively. Skewness has belonged to distortion or asymmetry in a symmetrical normal distribution in a dataset. If the curve is moved to the right or the left side, it is stated to be skewed. Also, skewness could be quantified as an impersonation of the range to which a given distribution differs from a normal distribution. For instance, the skew of zero value was measured for normal distribution, while, right skew is an indication of lognormal distribution [96]. The relationship between compressive strength and *l/b* with the Histogram of GGBS/FA-GPC mixtures at the age of 28 days is presented in Fig 2.

### b) Fly ash (*FA*)

**Fig 3:**
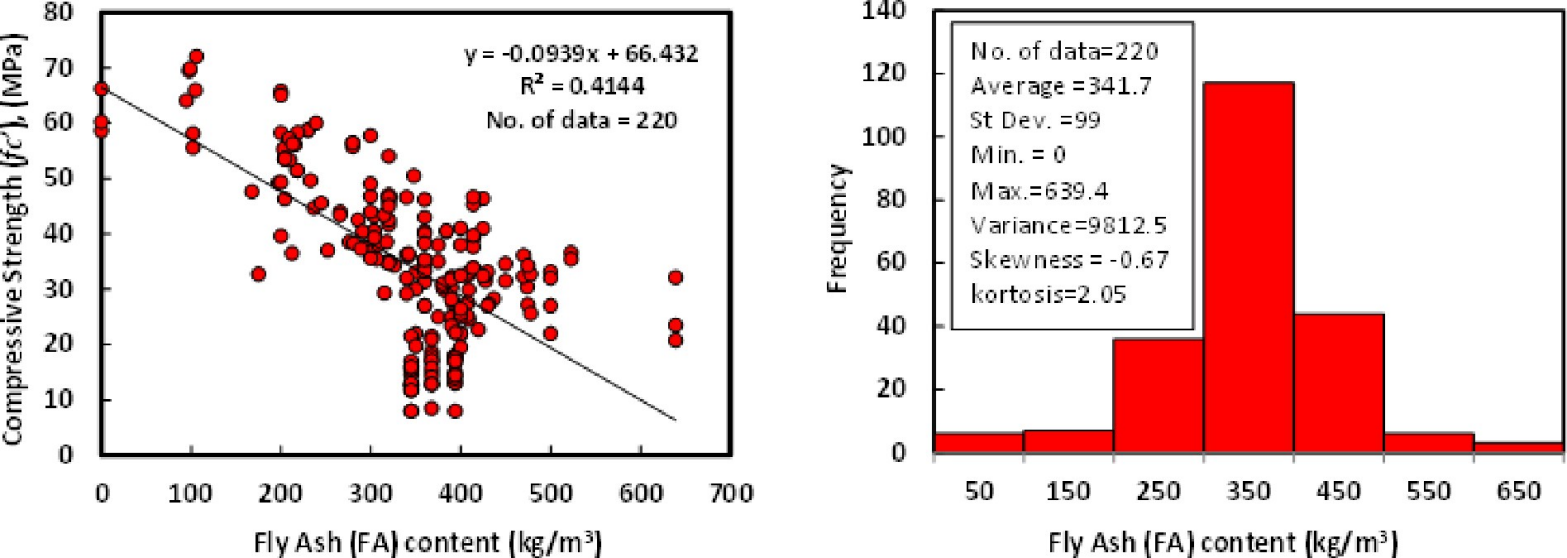
Relationship and a histogram of compression strength versus fly ash content of GGBS/FA-GPC.

The content of FA in the mixture proportions of different GGBS/FA-GPC for the collected data varied from 0 to 639.4 kg/m^3^. The FAs have different chemical compositions and different specific gravities, ranging from 1.92 to 2.55. The FA’s average, standard deviation, variance, skewness, and kurtosis were 341.7 kg/m^3^, 99 kg/m^3^, 9812.5, -0.67, and 2.05. The kurtosis is a statistical indicator that explains how heavily the tails of a distribution of a set of data differ from the tails of the normal distribution. In addition, the kurtosis finds the heaviness of the distribution tails, while skewness measures the symmetry of the distribution. Moreover, the variation between compressive strength and FA content and the Histogram of GGBS/FA-GPC mixtures at 28 days is reported in Fig 3.

### C) *Si/Al* of FA

**Fig 4:**
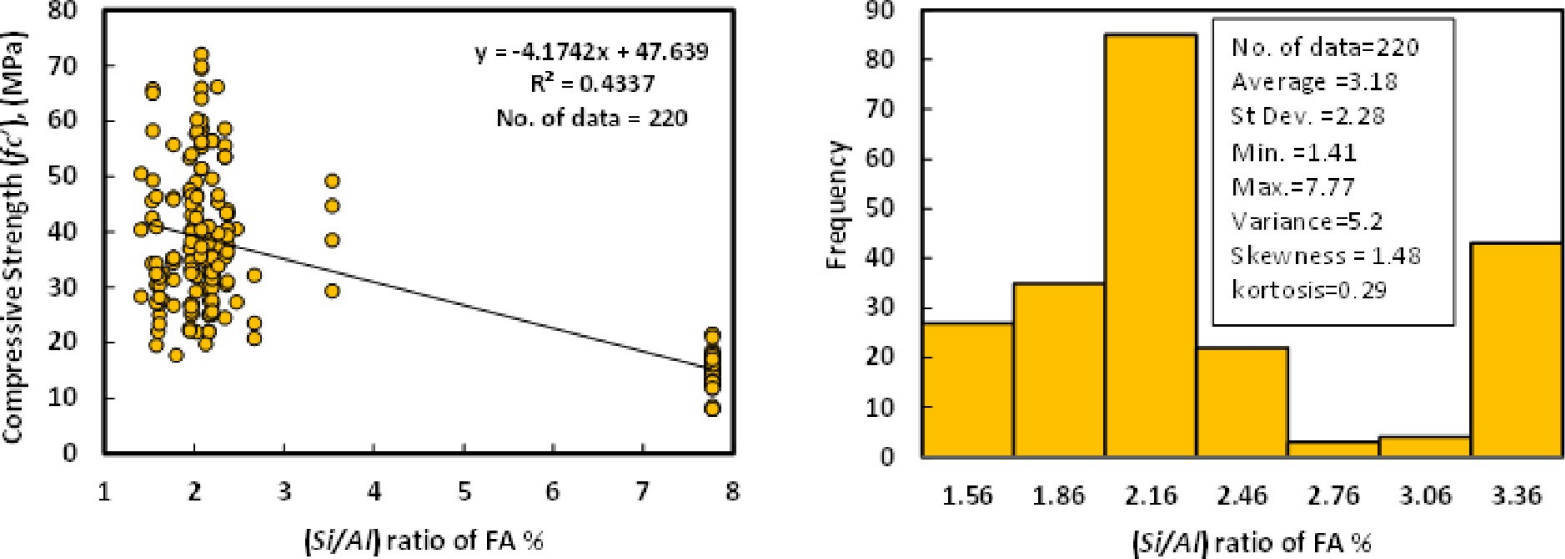
Relationship and a histogram of compression strength and Si/Al of fly ash.

Based on the dataset, which contains 220 data samples from literature, the *Si/Al* ratio of the fly ash was varied from 1.41 to 7.7 with an average of 3.18, the variance of 5.2, the standard deviation of 2.28 skewness of 1.48, and kurtosis of 0.29. The variance informed of the degree of spread in the dataset; the greater the spread of the data, the greater the variance is about the mean. The variation between compressive strength and *Si/Al*, as well as the histogram analysis of GGBS/FA-GPC at the age of 28 days, are shown in Fig 4. As can be seen from the figure, a very poor relationship existed between compressive strength and the *Si/Al* ratio.

### d) Ground granulated blast furnace slag (GGBS)

**Fig 5:**
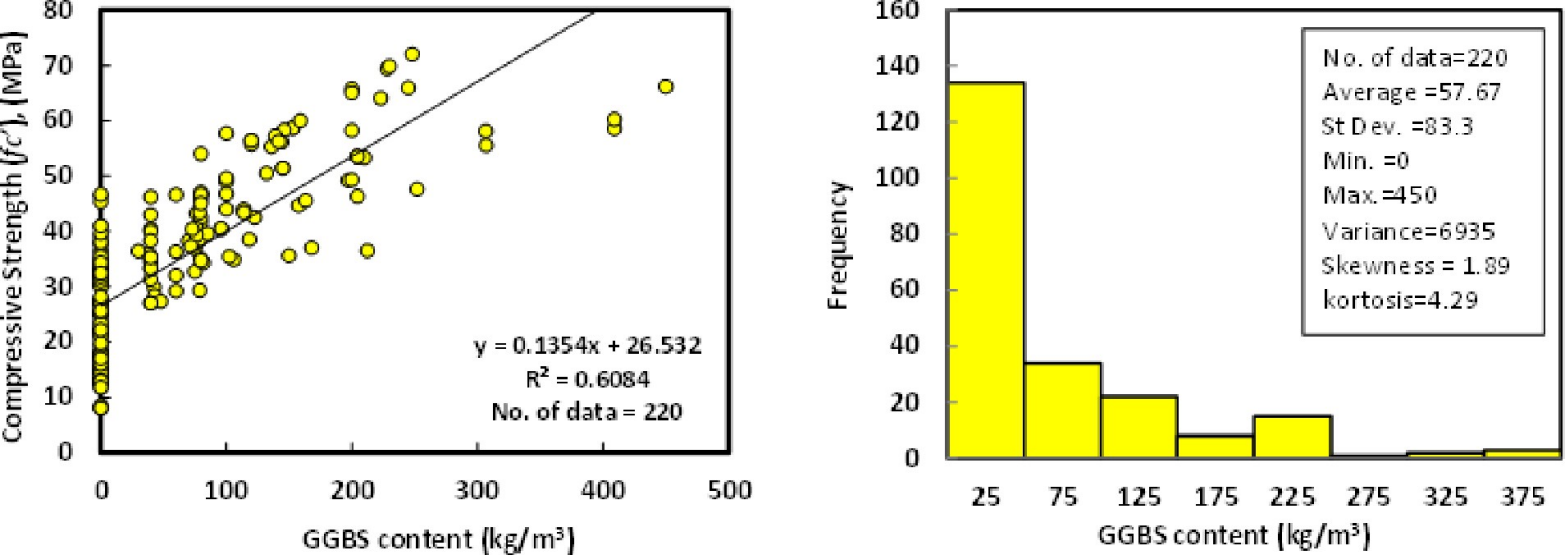
Relationship and a histogram of compression strength and curing temperature of GGBS/FA-GPC.

The statistical analysis for the total collected data of the 220 dataset shows that the range of the GGBS content was varied from 0 to 450 kg/m^3^, with an average of 57.67 kg/m^3^ and standard deviations of 83.3 kg/m^3^. Besides, the variance, skewness, and kurtosis were 6935, 1.89, and 4.29, correspondingly. The relationship between compressive strength and GGBS content with a Histogram of GGBS/FA-GPC mixtures at the age of 28 days is shown in Fig 5.

### e) SiO_2_/CaO (*Si/Ca*) of GGBS

**Fig 6:**
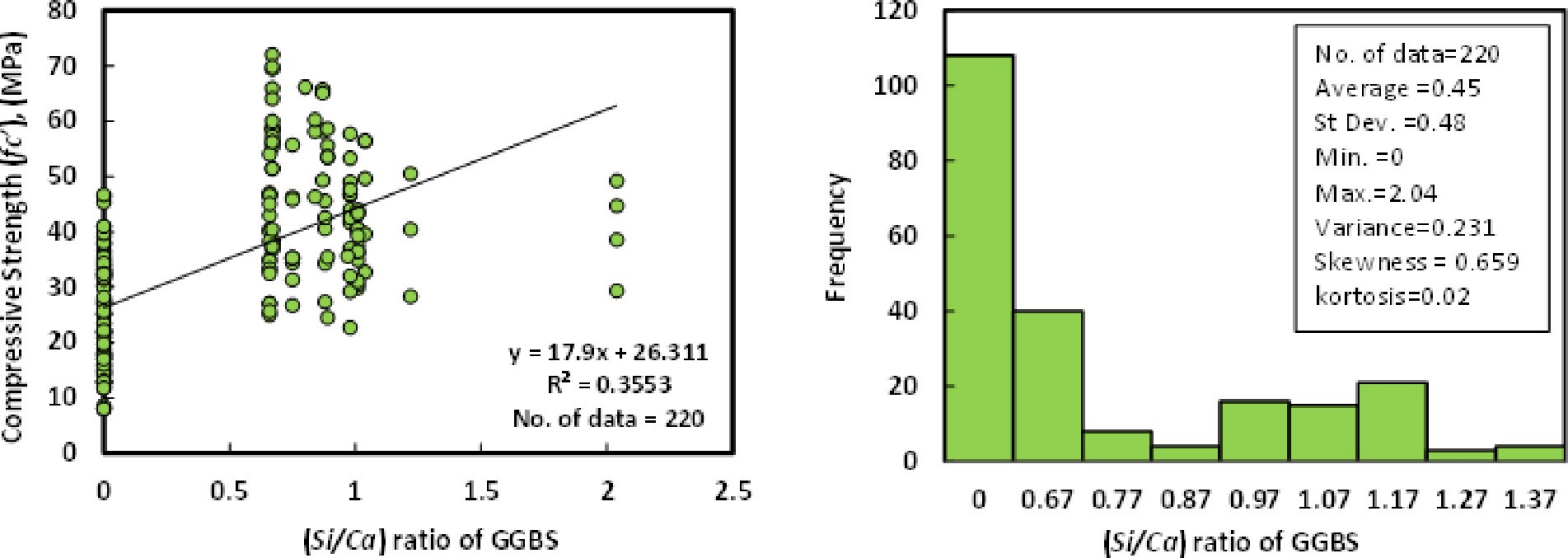
Relationship and a histogram of compression strength and curing duration of GGBS/FA-GPC.

Another independent variable gathered from previous research investigations was the GGBS’s silicon oxide to calcium oxide ratio. The statistical analysis indicated that the collected data set’s minimum Si/Ca was 0, and the maximum Si/Ca was 2.04. Furthermore, the average Si/Ca ratio was found to be 0.45. Fig 6 shows the relationship between compressive strength and the Si/Ca of GGBS/FA-GPC.

### f) Sand content (*F*)

**Fig 7:**
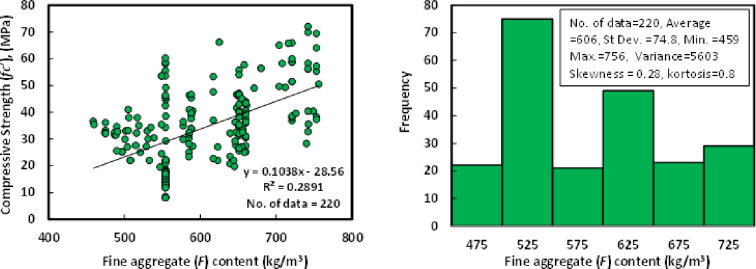
Relationship and a histogram of compression strength versus fine aggregate content of GGBS/FA-GPC.

The fine aggregate used in the past studies was a river and crushed sand with a maximum aggregate size of 4.75 mm and specific gravity ranging between 2.60–2.75. Also, its gradation satisfied the limitations of ASTM C 33. Fine aggregate content for the collected 220 datasets varied from 459 to 756 kg/m^3^ for the mixtures of GGBS/FA-GPC, and it has an average of 606 kg/m^3^, a standard deviation 74.8 kg/m^3^, a variance of 5603. Other statistical variables for the fine aggregate content in the GGBS/FA-GPC mixtures, such as skewness and kurtosis, are 0.28 and 0.8. The relationship between compressive strength and fine aggregate content with a Histogram of GGBS/FA-GPC mixtures at the age of 28 days is illustrated in Fig 7.

### G) Coarse aggregate (*C*)

**Fig 8:**
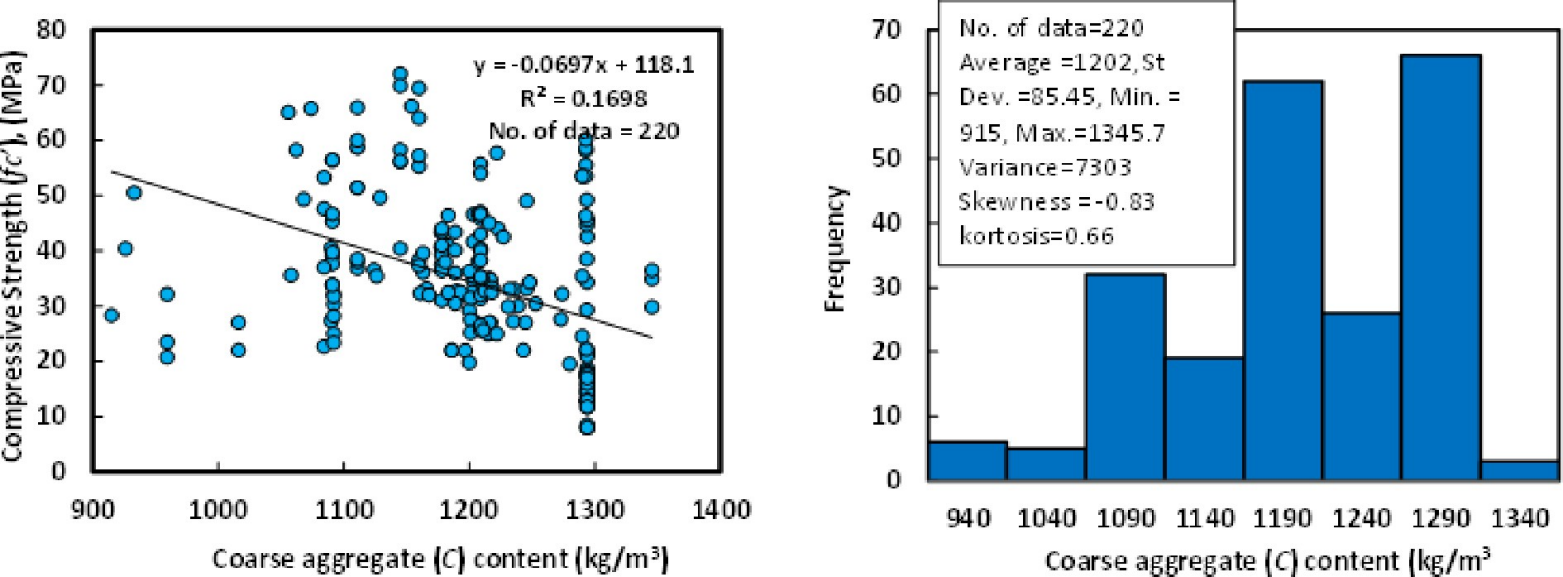
Relationship and a histogram of compression strength versus coarse aggregate content of GGBS/FA-GPC.

Crushed stone or gravel with a maximum aggregate size of 20 mm was used as coarse aggregate for GGBS/FA-GPC in the literature. Based on the 220 datasets available from various GGBS/FA-GPC admixture proportions, the coarse aggregate concentration varied from 915 to 1345.7 kg/m3. According to the statistical analysis of the present dataset, the average coarse aggregate content was 1202 kg/m3. The skewness was -0.83, and the kurtosis was 0.66. The range was 7303, and the skewness was -0.83. The variance was 7303, the skewness was -0.83, and the kurtosis was 0.66. The standard deviation was 85.45 kg/m^3^, the variance was 7303, the skewness was -0.83, and the kurtosis was 0.66. Fig 8 uses a Histogram of GGBS/FA-GPC mixtures at 28 days to demonstrate the connection between compressive strength and coarse aggregate content.

### h) Sodium hydroxide (*SH*)

**Fig 9:**
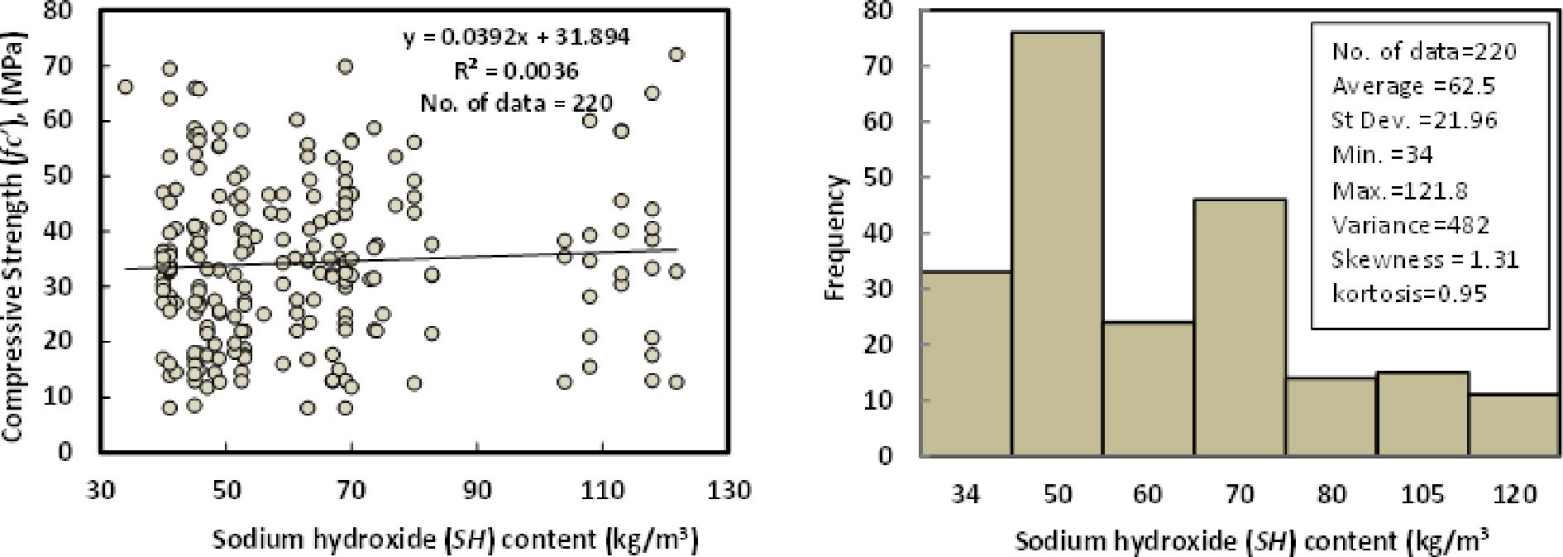
Relationship and a histogram of compression strength and sodium hydroxide content of GGBS/FA-GPC.

The sodium hydroxide (NaOH) content for the collected 220 datasets varied from 34 to 121.8 kg/m3, with an average of 62.5 kg/m3, the standard deviation of 21.96 kg/m3, and a variance of 482. The skewness and kurtosis were 1.31 and 0.95, respectively. The purity of the SH was above 97% of all the GGBS/FA-GPC mixtures, and pellets and flakes were the two main states of the SH in all the mixtures. The relationship between compressive strength and sodium hydroxide with the Histogram of GGBS/FA-GPC mixtures at the age of 28 days is illustrated in Fig 9.

### i) Sodium silicate (*SS*)

**Fig 10:**
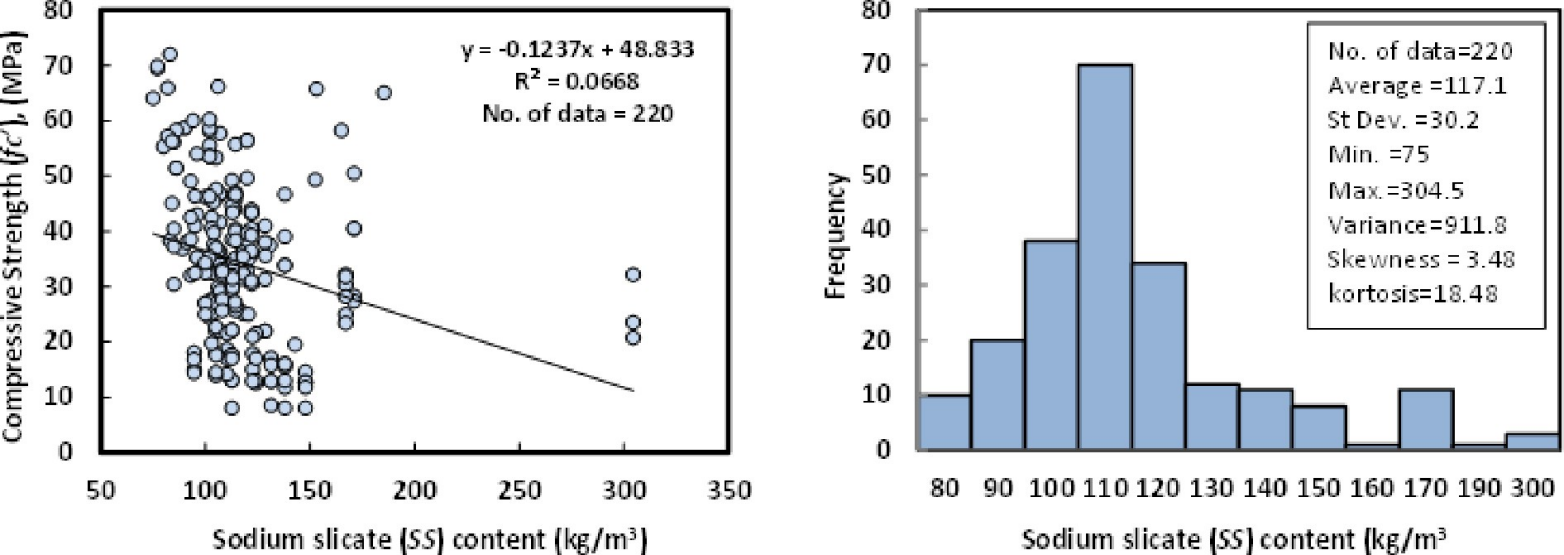
Relationship and a histogram of compression strength and sodium silicate content of GGBS/FA-GPC.

The amount of SS was changed between 75 and 304.5 kg/m3 based on the dataset, which comprises 220 data samples from literature. SiO2, Na2O, and water were the main components of the SS. SiO2 concentrations ranged from 28 to 37 percent, Na2O concentrations from 8 to 18 percent, and water content in the SS from 45 to 64 percent. The average SS content in the GGBS/FA-GPC was 117.1 kg/m^3^, the standard deviation was 30.2 kg/m^3^, the variance was 911.8, the skewness was 3.48, and the kurtosis was 18.48, according to the statistical analysis (Fig 10).

### j) SS/SH

**Fig 11:**
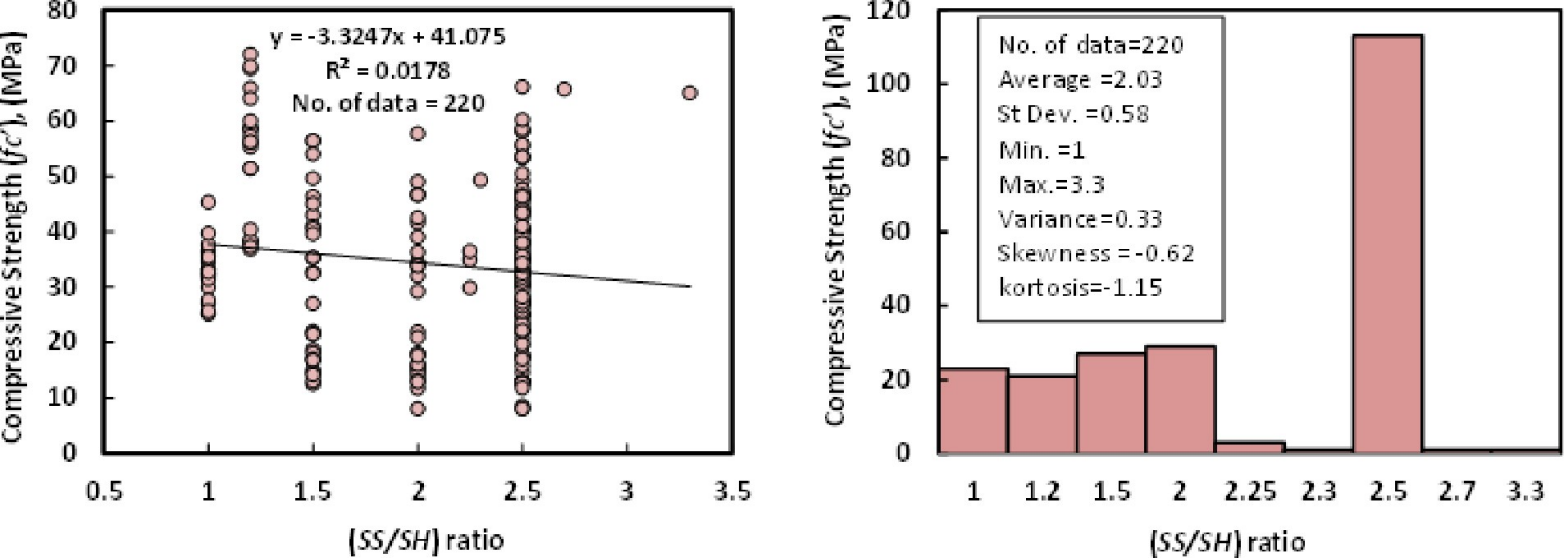
Relationship and a histogram of compression strength and (SS/SH) ratio of GGBS/FA-GPC.

The ratio of Na_2_SiO_3_ to NaOH in the obtained data ranged from 1 to 3.3, with an average of 2.03. Standard deviation, variance, skewness, and kurtosis, respectively, were 0.58, 0.33, -0.62, and -1.15. Fig 11 shows the connection between compressive strength and SS/SH with the Histogram of GGBS/FA-GPC mixes after 28 days.

### k) Molarity (*M*)

**Fig 12:**
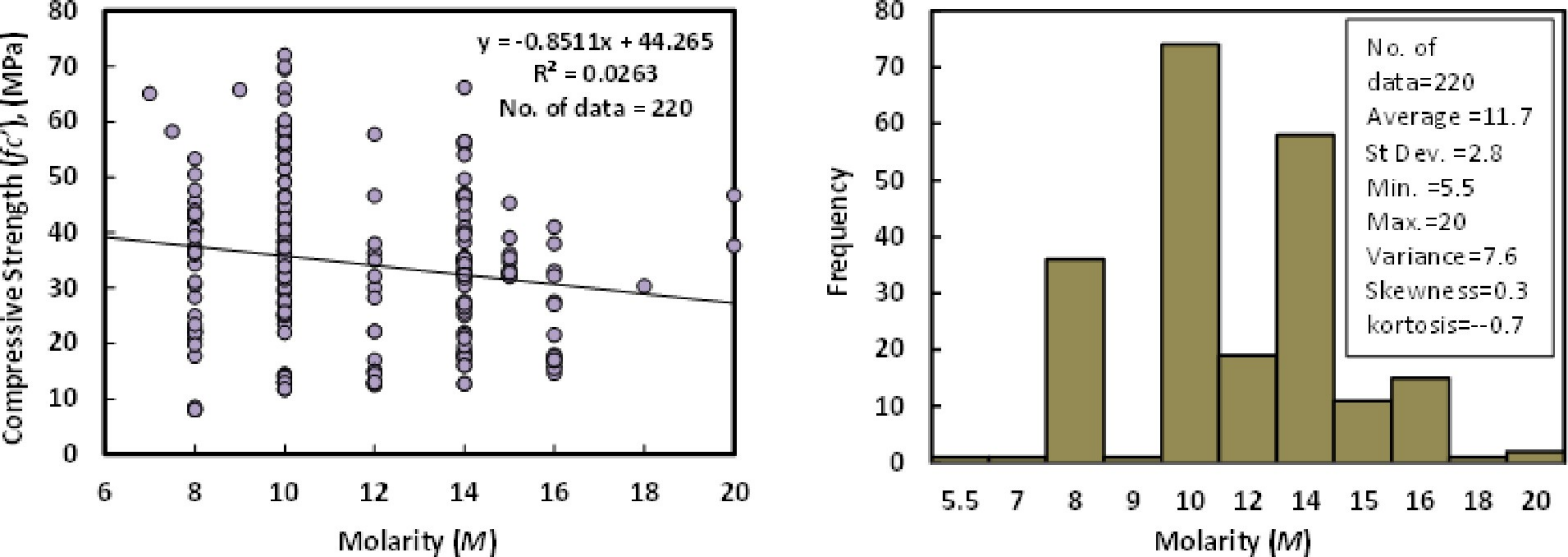
Relationship and a histogram of compression strength and molarity with a histogram of GGBS/FA-GPC mixtures.

The sodium hydroxide concentration (molarity) ranged from 5.5 to 20 M in 220 datasets from prior research investigations (Table 1), with an average of 11.7 M, a standard deviation of 2.8 M, a variance of 7.6, a skewness of 0.3, and kurtosis of -0.7. Fig 12 depicts the relationship between compressive strength and molarity of GGBS/FA-GPC after 28 days.

### l) Compressive strength (*fc′*)

The compression strength of the GGBS/FA-GPC was tested in the range of 8 to 72 MPa, with an average of 34.34 MPa, according to data obtained from published research (Table 1). According to the statistical analysis, the standard deviation, variance, skewness, and kurtosis for the other dataset distribution were 14.45 MPa, 208.84, 0.32, and -0.47, respectively.

## 4. Modeling

The models proposed in this work are used to predict GGBS/FA-GPC and choose the best solution that provides a better compressive strength estimate than the experimentally obtained compressive strength. The datasets were divided into three categories at random: training, testing, and validating datasets [48, 60]. The LR, MLR, ANN, and M5P-tree models are trained with 150 training datasets to get the best weights and biases, while the suggested models are tested with 35 testing datasets. Furthermore, 35 validation datasets are utilized to investigate the models’ generality and prevent the over-fitting problem plaguing traditional training techniques. The following evaluation criteria were used to compare model predictions: The model should be scientifically correct, with reduced RMSE, OBJ, SI, and R^2^ values, as well as a decreased fraction of error between observed and predicted data.

### a) Linear model (LR)

One of the most common methods to predict the compressive strength of concrete is the linear regression model (LR) [95–97], as shown in *Eq*.*1*, and it is considered as a general form of the linear regression model [57, 60, 96].


fc′=a+b(l/b) (1)

Where *fc′*, lb, *a and b* represents compressive strength, liquid to binder ratio, and equation parameters, respectively. However, other components of GGBS/FA-GPC mixtures that influence the compressive strength, such as fly ash content and GGBS content, and other mix proportions, are not included in the equation above. Therefore, in order to have more reliable and scientific observations, *Eq*.*2* is proposed to include all other mix proportions and variables that may impact the compressive strength of GGBS/FA-GPC.


$$fc\prime =a+b(\frac{l}{b})+c(FA)+d(\frac{Si}{Al})+e(GGBS)+f(\frac{Si}{Ca})+g(F)+h(C)+i(SH)+j(SS)+k(\frac{SS}{SH})+l(M)$$ (2)

Where: *l/b* is the alkaline solution to the binder ratio, *FA* is the fly ash (kg/m^3^) content, *Si/Al* is the ratio of SiO_2_ to Al_2_O_3_ of the fly ash, *GGBS* is the ground granulated blast furnace slag (kg/m^3^) content, *Si/Ca* is the ratio of SiO_2_/CaO of GGBS, *F* is the fine aggregate (kg/m^3^) content, *C* is the coarse aggregate (kg/m^3^) content, *SH* is the sodium hydroxide (kg/m^3^) content, *SS* is the sodium silicate (kg/m^3^) content, *SS/SH* is the ratio of sodium silicate to the sodium hydroxide, and *M* is the sodium hydroxide concentration (Molarity). While *a*, *b*, *c*, *d*, *e*, *f*, *g*, *h*, *i*, *j*, *k*, and *l* are the model parameters. This developed equation is a unique equation that involves a wide range of independent variables to produce GGBS/FA-GPC that may be very useful for the construction industry. The proposed *Eq*.*2* can be considered as an extent for *Eq*.*1* since all variables can be adapted linearly.

### b) Multi-logistic regression model (MLR)

Same as the former models, the multi-logistic regression analysis model was carried out for the collected datasets. The general form of the MLR is shown in Eq 3 based on the research studied conducted by Mohammed et al. [57] and Faraj et al. [60]. MLR is used to clarify the difference between a nominal predictor variable and one or more independent variables.


$$fc\prime =a*{\left(\frac{l}{b}\right)}^{b}*({FA)}^{c}*{\left(\frac{Si}{Al}\right)}^{d}*({GGBS)}^{e}*{\left(\frac{Si}{Ca}\right)}^{f}*{\left(F\right)}^{g}*{\left(C\right)}^{h}*{\left(SH\right)}^{i}*{\left(SS\right)}^{j}*{\left(\frac{SS}{SH}\right)}^{k}*{\left(M\right)}^{l}$$ (3)

Where: *l/b* is the alkaline solution to the binder ratio, *FA* is the fly ash (kg/m^3^) content, *Si/Al* is the ratio of SiO_2_ to Al_2_O_3_ of the fly ash, *GGBS* is the ground granulated blast furnace slag (kg/m^3^) content, *Si/Ca* is the ratio of SiO_2_/CaO of *GGBS*, *F* is the fine aggregate (kg/m^3^) content, *C* is the coarse aggregate (kg/m^3^) content, *SH* is the sodium hydroxide (kg/m^3^) content, *SS* is the sodium silicate (kg/m^3^) content, *SS/SH* is the ratio of sodium silicate to the sodium hydroxide, and *M* is the sodium hydroxide concentration (Molarity). While *a*, *b*, *c*, *d*, *e*, *f*, *g*, *h*, *i*, *j*, *k*, and *l* are the model parameters.

### c) Artificial neural network model (ANN)

ANN is a powerful simulation software designed for data analysis and computation to think like a human brain in processing and analyses. This machine learning tool is widely used in construction engineering to predict several numerical problems’ future behavior [51, 98, 99]. ANN model is generally divided into three main layers: input, hidden, and output layers. Each input and output layer can be one or more layers depending on the proposed problem. However, the hidden layer is usually ranged for two or more layers. Although the input and output layers are generally depending on the collected data and the designed model purpose, the hidden layer is determined by rated weight, transfer function, and the bias of each layer to other layers. A multi-layer feed-forward network is built based on a mixture of proportions, weight/bias, several parameters, including (*l/b*, *F*, *Si/Al*, *GGBS*, …) as inputs, and output ANN here is compressive strength. There is no standard approach to designing the network architecture. Therefore, the number of hidden layers and neurons is determined based on a trial and error test. One of the main objectives of the training process of the network is to determine the optimum number of iterations (epochs) that provide the minimum mean absolute error (MAE), root mean square error (RMSE), and best R-value that close to one. The effect of several epochs on reducing the MAE and RMSE has been studied.

The collected data set (a total of 220 data) has been divided into three parts for the training purpose of the designed ANN. Around 70^th^ percent of the collected data was used as trained data for training the network. The 15th percent of overall data was used to test the data set, and the rest of the remaining data was used to validate the trained network [98]. The designed ANN was trained and tested for various hidden layers to determine optimal network structure based on the fitness of the predicted compressive strength of GGBS/FA-GPC with the compressive strength of the real collected data. It was observed that the ANN structure with two hidden layers, twelve neurons, and a hyperbolic tangent transfer function was a best-trained network that provides a maximum R^2^ and minimum both MAE and RMSE (shown in Table 2). As a part of this study, an ANN model has been used to predict the future value of the compressive strength of GGBS/FA-GPC. The general equation of the ANN model is shown in *Eq*.*4*, *Eq*.*5*, and *Eq*.*6*.

From linear node 0:

$$fc^{\prime }=Threshold+\left(\frac{Node\;1}{1+e^{-B1}}\right)+\left(\frac{Node\;2}{1+e^{-B2}}\right)+\cdots$$ (4)

From sigmoid node 1:

B1=Threshold+Σ(Attribute*Variable) (5)

From sigmoid node 2:

B2=Threshold+Σ(Attribute*Variable) (6)

**Table 2:** The tested ANN architectures.

No. of (HL) Hidden layers	No. of Neurons	R^2^	MAE (MPa)	RMSE (MPa)
1	1	0.9528	4.28	5.36
1	2	0.9648	3.09	4.06
1	3	0.9742	3.28	4.05
1	4	0.9792	2.42	3.14
1	5	0.9846	2.49	3.14
1	6	0.9855	2.75	3.37
1	7	0.9851	2.8406	3.5044
1	8	0.9829	3.82	4.5525
2	2	0.9503	4.4414	5.6781
2	3	0.9516	4.4791	5.6041
2	3	0.9679	3.3809	4.3152
2	4	0.9708	2.7214	3.623
2	5	0.9684	3.311	4.2624
2	5	0.9747	3.1049	3.8942
2	6	0.9713	2.9421	3.7988
2	7	0.9703	3.222	4.0899
2	7	0.9794	2.9247	3.5783
2	8	0.9776	3.1316	3.9178
2	9	0.9728	3.3017	4.0494
2	10	0.9813	2.5421	3.2451
2	11	0.9796	2.5518	3.2376
**2**	**12**	**0.9881**	**1.8723**	**2.478**
2	13	0.9785	3.0593	3.7905
2	14	0.981	2.6014	3.2927
2	18	0.9795	3.4181	4.0444
2	24	0.9788	3.1395	3.9741
2	13	0.9783	3.6006	4.3096
2	11	0.9785	3.5253	4.2822
2	10	0.9791	3.4389	4.1613

### d) M5P-tree model

The M5P model tree is a reconstruction of Quinlan’s M5P-tree algorithm [100] that is based on the conventional decision tree with the addition of a linear regression function to the leaves nodes. The decision tree represents the algorithms by a tree form trained through data to form nodes. The decision tree nodes are divided into root, internal, and leaves nodes. Nodes are interconnected through branches until the leaves are reached [101–107]. Furthermore, M5P-tree, introduced by [99], is a robust decision tree learner model to study regression analysis. This learner algorithm puts linear regression functions at the terminal nodes. It places a multivariate linear regression model to each sub-space by classifying all data sets into multiple sub-spaces. The M5P-tree works on continuous class problems rather than discrete segments and can handle tasks with high dimensional features. It reveals the developed information of each linear model component constructed to estimate the nonlinear correlation of the data sets. The information about division criteria for the M5-tree model is obtained through the error calculation at each node. At each node, errors are analyzed by the standard deviation of the class entering that node. The attribute that maximizes the reduction of estimated error at each node is used to evaluate any task of that node. As a result of this division in the M5P tree, a large tree-like structure that leads to overfitting will be created. The enormous tree is trimmed in the followed step, and linear regression functions restore the pruned subtrees. The general equation form of the M5P-tree model is the same as the linear regression equation, as shown in *Eq*. *7*

$$fc^{\prime }=a+b(\frac{l}{b})+c(FA)+d(\frac{Si}{Al})+e(GGBS)+f(\frac{Si}{Ca})+g(F)+h(C)+i(SH)+j(SS)+k(\frac{SS}{SH})+l(M)$$ (7)

## 5. Model performance assessment criteria


$${coefficient\;of\;determination,R}^{2}={\left(\frac{\sum_{p=1}^{p}\left(t_{p}-t^{\prime })(y_{p}-y^{\prime }\right)}{\sqrt{\left[\sum_{p=1}^{p}{\left(t_{p}-t^{\prime }\right)}^{2}][\sum_{p=1}^{p}{\left(y_{p}-y^{\prime }\right)}^{2}\right]}}\right)}^{2}$$ (8)

$$Root\;Mean\;Squared\;Error,\;RMSE=\sqrt{\frac{\sum_{p=1}^{p}{\left(y_{p}-t_{p}\right)}^{2}}{n}}$$ (9)

$$Mean\;Absolute\;Error,\;MAE=\frac{\sum_{p=1}^{p}\left|(y_{p}-t_{p})\right|}{n}$$ (10)

$$SI=\frac{RMSE}{t^{\prime }}\;,Scatter\;Index$$ (11)

$$OBJ=\left(\frac{n_{tr}}{n_{all}}*\frac{{{RMSE}_{tr}+MAE}_{tr}}{R_{tr}^{2}+1}\right)+\left(\frac{n_{tst}}{n_{all}}*\frac{{{RMSE}_{tst}+MAE}_{tst}}{R_{tst}^{2}+1}\right)+\left(\frac{n_{val}}{n_{all}}*\frac{{{RMSE}_{val}+MAE}_{val}}{R_{val}^{2}+1}\right)$$ (12)

Where: ***y***_***p***_ and ***t***_***p***_ are the estimated and the tested compressive strength values, and ***t′*** and ***y′*** are the averages of the experimentally tested and the predicted values from the models, respectively. ***tr***, ***tst*,** and ***val*** are training, testing, and validating datasets, respectively, and ***n*** is the number of datasets.

Except for the R^2^ value, zero is the optimal value for all other evaluation parameters. However, one is the highest benefit for R^2^. When it comes to the SI parameter, a model has bad performance when it is > 0.3, acceptable performance when it is 0.2 SI 0.3, excellent performance when it is 0.1 SI 0.2, and great performance when it is 0.1 SI 0.1 [48, 60, 103]. Furthermore, the OBJ parameter was employed as a performance measurement parameter in Eq 12 to measure the efficiency of the suggested models.

## 6. Results and analysis

### a) LR model

**Fig 13:**
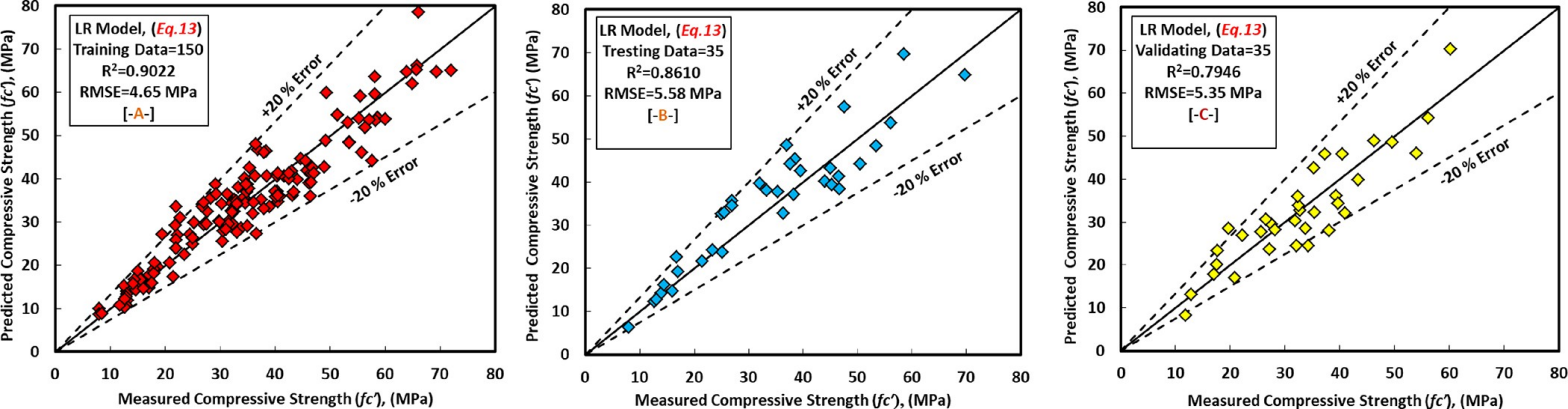
Comparison between measured and predicted compressive strength of GGBS/FA-GPC mixture using LR model, (a) training data, (b) testing data, (c) validating data.

Based on the linear regression analysis model, it was discovered that the l/b, SS/SH, and M of the GC mixture have a significant impact on the compressive strength of the GGBS/FA-GPC, which is consistent with experimental data published in the literature. [20, 22, 66, 92, 104–107]. The equation for the LR model with different weight parameters can be written as follows (Eq 13) and Fig 13.


$$fc^{\prime }=148.56-120.3(\frac{l}{b})-0.179(FA)-2.24(\frac{Si}{Al})-0.074(GGBS)+3.33(\frac{Si}{Ca})-0.0018(F)-0.0289(C)+0.2734(SH)+0.2218(SS)-3.779(\frac{SS}{SH})+0.992(M)$$ (13)

**Fig 14:**
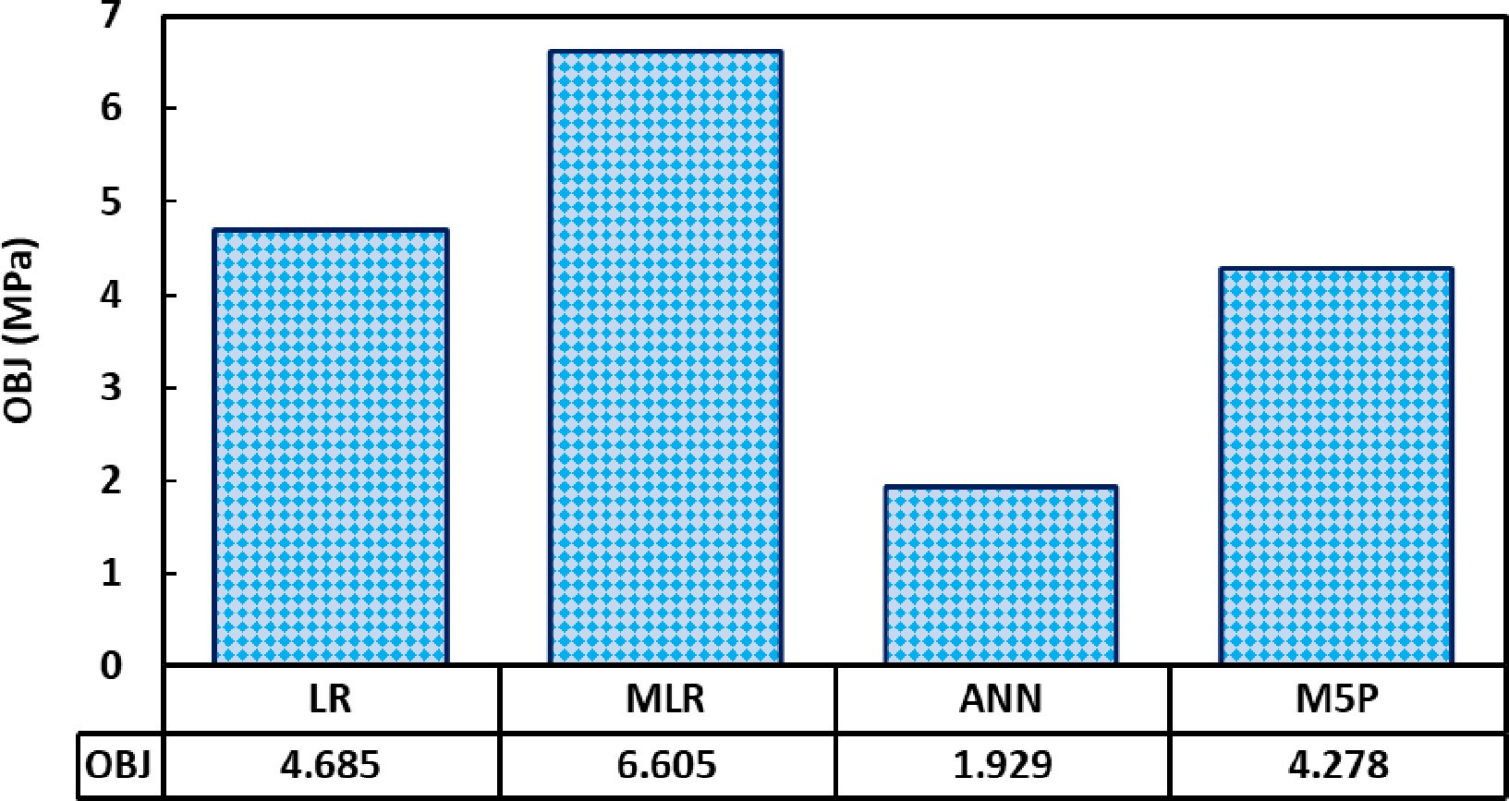
Barchart of OBJ values of the models used.

**Fig 15:**
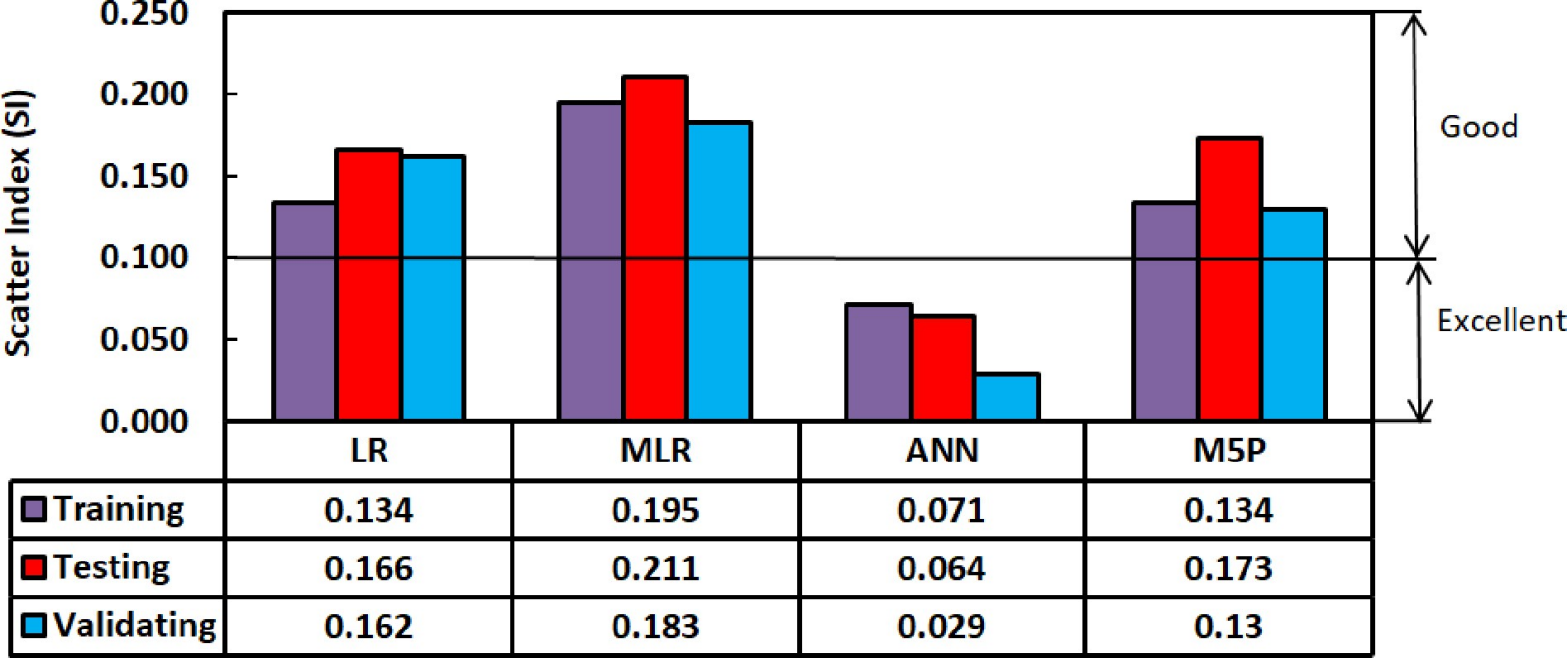
Scatter index (SI) performance for the models used in this study.

**Fig 16:**
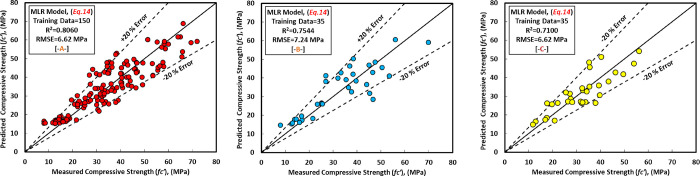
Comparison between measured and predicted compressive strength using MLR model, (a) training data, (b) testing data, and (c) validating data.

For all of the training, testing, and validation datasets, there is a 20 percent error line. Nonetheless, the generated model somewhat underestimated GGBS/FA-GPC and slightly overestimated low strength GGBS/FA-GPC mixtures. In addition, utilizing the training, testing, and validating datasets, the residual compressive strength between the predicted and observed compressive strength for the LR model was evaluated, as shown in Fig 14. The R^2^, RMSE, and MAE evaluation variables for this model are the same as 0.9022, 4.65 MPa, and 3.60 MPa. Furthermore, as shown in Figs 15 and 16, the current model’s OBJ and SI values for the training dataset are 2.96 and 0.134, respectively.

### b. MLR model

Eq 14 shows the generated models for the MLR model with various variable parameters. The most significant independent factors that impact the compressive strength of the GGBS/FA-GPC in the MLR model were replacement level of GGBS content, SS content, and SS/SH, which are matched with experimental studies published in the literature [22, 32, 33, 61, 62, 73, 75, 86, 92]. Fig 16(A) depicts the correlations between anticipated and measured compressive strength in the GGBS/FA-GPC training data set. Furthermore, similar to the previous models, this model was tested using two sets of data (testing and validating datasets) to demonstrate its efficacy for variables not included in the model data (training data). The findings indicate that by substituting the independent variables into the established equation, this model can predict the compressive strength of GGBS/FA-GPC, as illustrated in Fig 16(B) and 16(C).


$$fc\prime =643.334*{\left(\frac{l}{b}\right)}^{-0.5}*({FA)}^{-0.086}*{\left(\frac{Si}{Al}\right)}^{-0.329}*{\left(GGBS\right)}^{0.174}*{\left(\frac{Si}{Ca}\right)}^{-0.002}*{\left(F\right)}^{-0.147}*{\left(C\right)}^{-0.4}{*(SH)}^{-9.22}*{\left(SS\right)}^{9.5}*{\left(\frac{SS}{SH}\right)}^{-9.53}*{\left(M\right)}^{0.084}$$ (14)

The examined datasets, like another model, contain a 20% error line for all training, testing, and validating datasets, suggesting that nearly all tested findings were within 20% error lines. Finally, utilizing training, testing, and validating datasets, the residual compressive strength for the MLR model for predicted and observed compressive strength was displayed in Fig 14.

### C) ANN model

**Fig 17:**
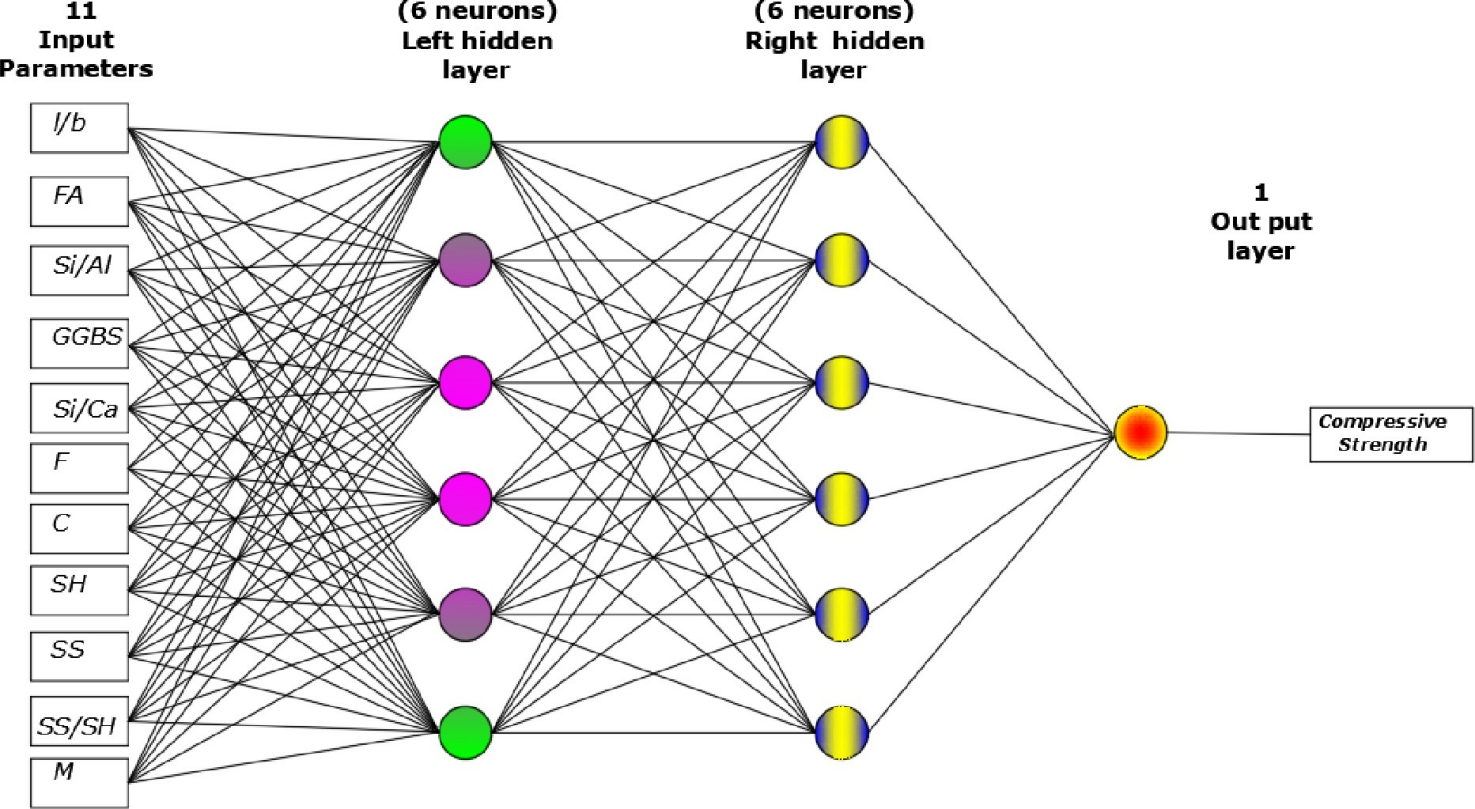
Optimal network structures of the ANN Model.

**Fig 18:**
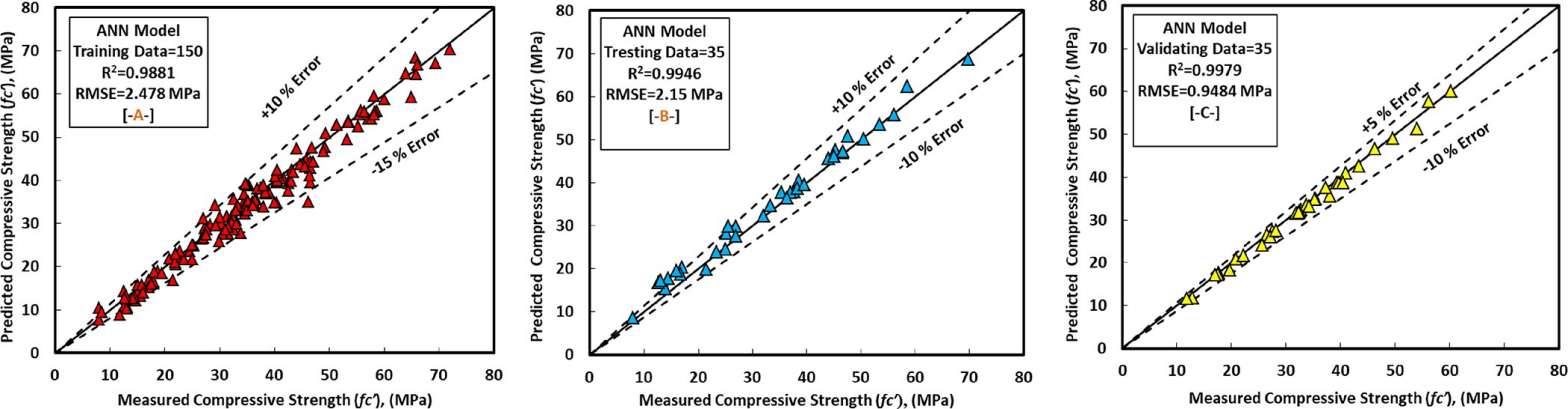
Measured-predicted compressive strength of GGBS/FA-GPC using ANN model, (a) training data, (b) testing data, and (c) validating data.

**Fig 19:**
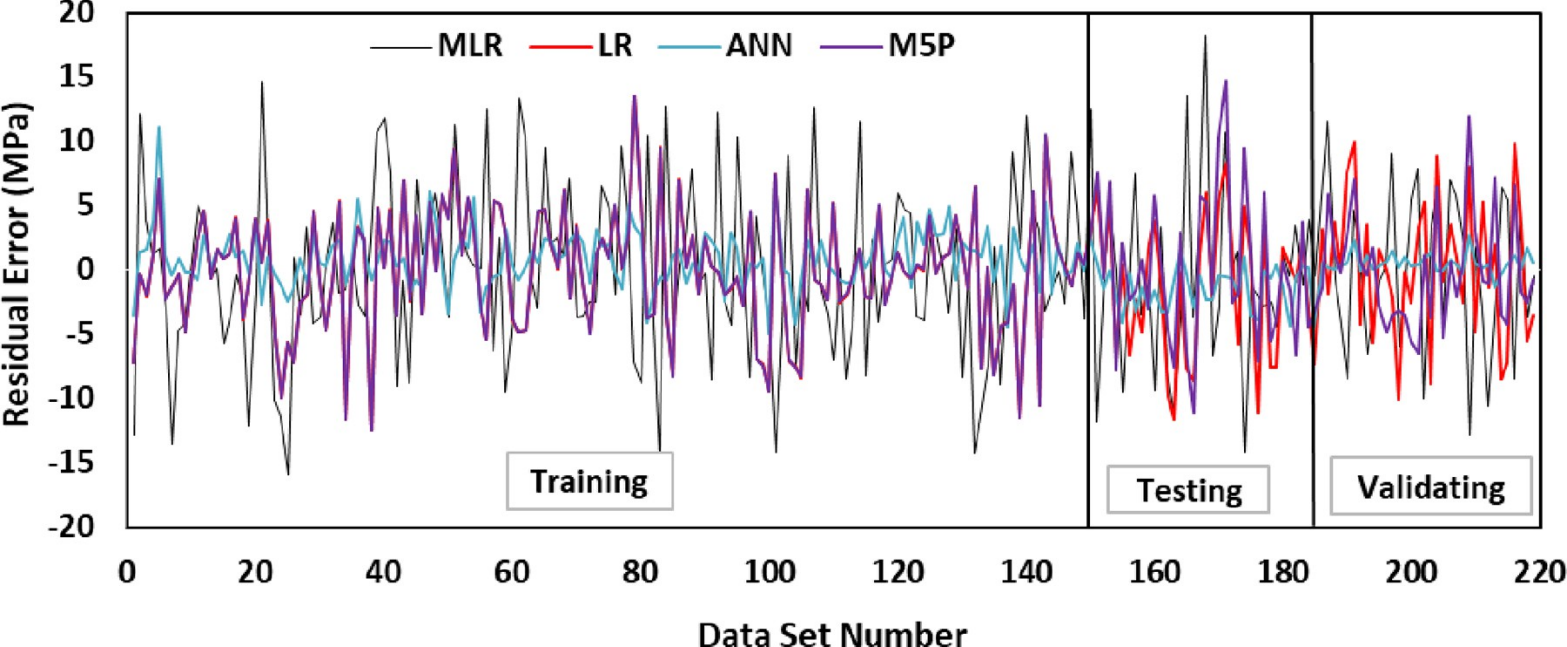
Relationship between Residual error and compressive strength of GGBS/FA-GPC mixtures.

In this study, the authors tried a lot to get the high efficiency of the ANN by applying different numbers of the hidden layer, neurons, momentum, learning rate, and iteration. Lastly, they observed that when the ANN has two hidden layers, 12 neurons (6 for the left side and 6 for the right side as shown in Fig 17, 0.2 momentum, 0.1 learning rate, and 2000 iteration give best-predicted values of the compressive strength of the GGBS/FA-GPC. The ANN model was equipped with the training datasets, testing and validating datasets to predict the compression strength values for the correct input parameters. The comparison between predicted and measured compressive strengths of GGBS/FA-GPC for training, testing, and validating datasets are presented in Fig 18(A)–18(C). The studied datasets have a +10% and -15% error line for the training data, ±10% error lines for testing data, and +5% and -10% for the validating datasets, which is better than the other developed models. Furthermore, this model has a better performance compared to other models to predict the compressive strength of GGBS/FA-GPC based on the value of OBJ and SI that illustrated in Figs 14 and 15, also, the value of R^2^ = 0.9881, MAE = 1.8723 MPa, and RMSE = 2.478 MPa. Finally, the residual compressive strength for the ANN model was shown in Fig 19 for the predicted and measured compressive strength by using training, testing, and validating datasets.

### d) M5P-tree model

**Fig 20:**
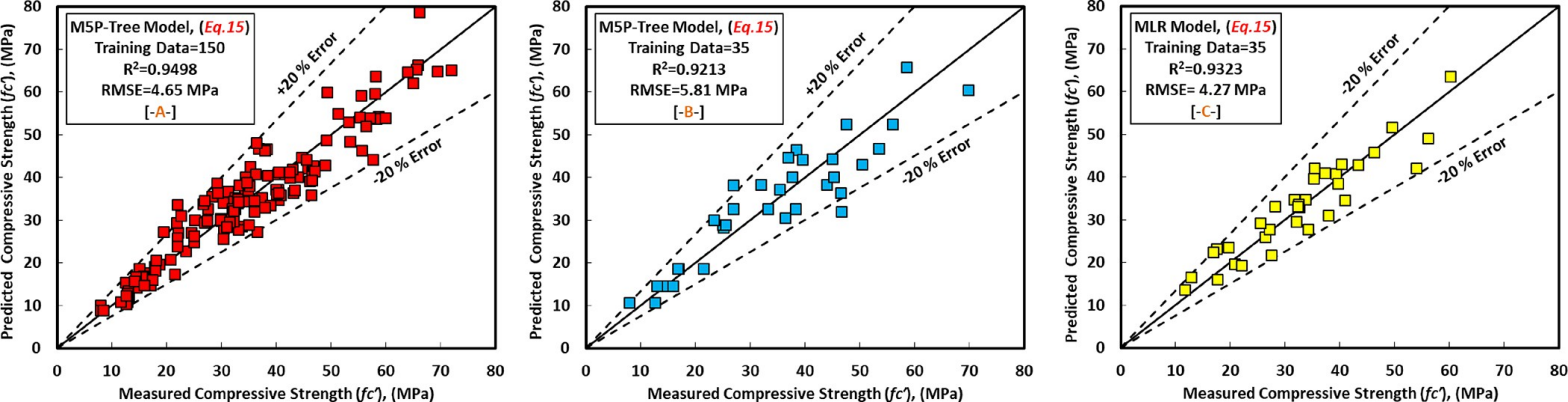
Comparison between measured and predicted compressive strength of GGBS/FA-GPC mixture using M5P model, (a) training data, (b) testing data, (c) validating data.

The predicted and observed compressive strengths of GGBS/FA-GPC for training, testing, and validating datasets are shown in Fig 20(A)–20(C). Similar to other models, it was discovered that the alkaline liquid to binder ratio (l/b) and the sodium silicate to sodium hydroxide ratio of the GGBS/FA-GPC mixture has a significant impact on the compressive strength of the GGBS/FA-GPC, which agrees with experimental findings in the literature [66, 104–107]. The model parameters are provided in Eq 15, and the model variables will be chosen using the linear tree registration function.


$$fc^{\prime }=145.1378-119.7338(\frac{l}{b})-0.1773(FA)-2.2382(\frac{Si}{Al})-0.0729(GGBS)+3.2516(\frac{Si}{Ca})-0.028(C)+0.2796(SH)+0.2178(SS)-3.5201(\frac{SS}{SH})+0.9866(M)$$ (15)

For all of the training, testing, and validation datasets, there is a 20 percent error line. Finally, for all datasets, the residual compressive strength for the M5P model was displayed in Fig 19 for both predicted and observed compressive strength. Furthermore, this model’s R2, RMSE, MAE, OBJ, and SI evaluation criteria are 0.9498, 4.6557 MPa, 3.7864 MPa, 4.278, and 0.134, respectively, for the training datasets.

## 6. Developed models performance

**Fig 21:**
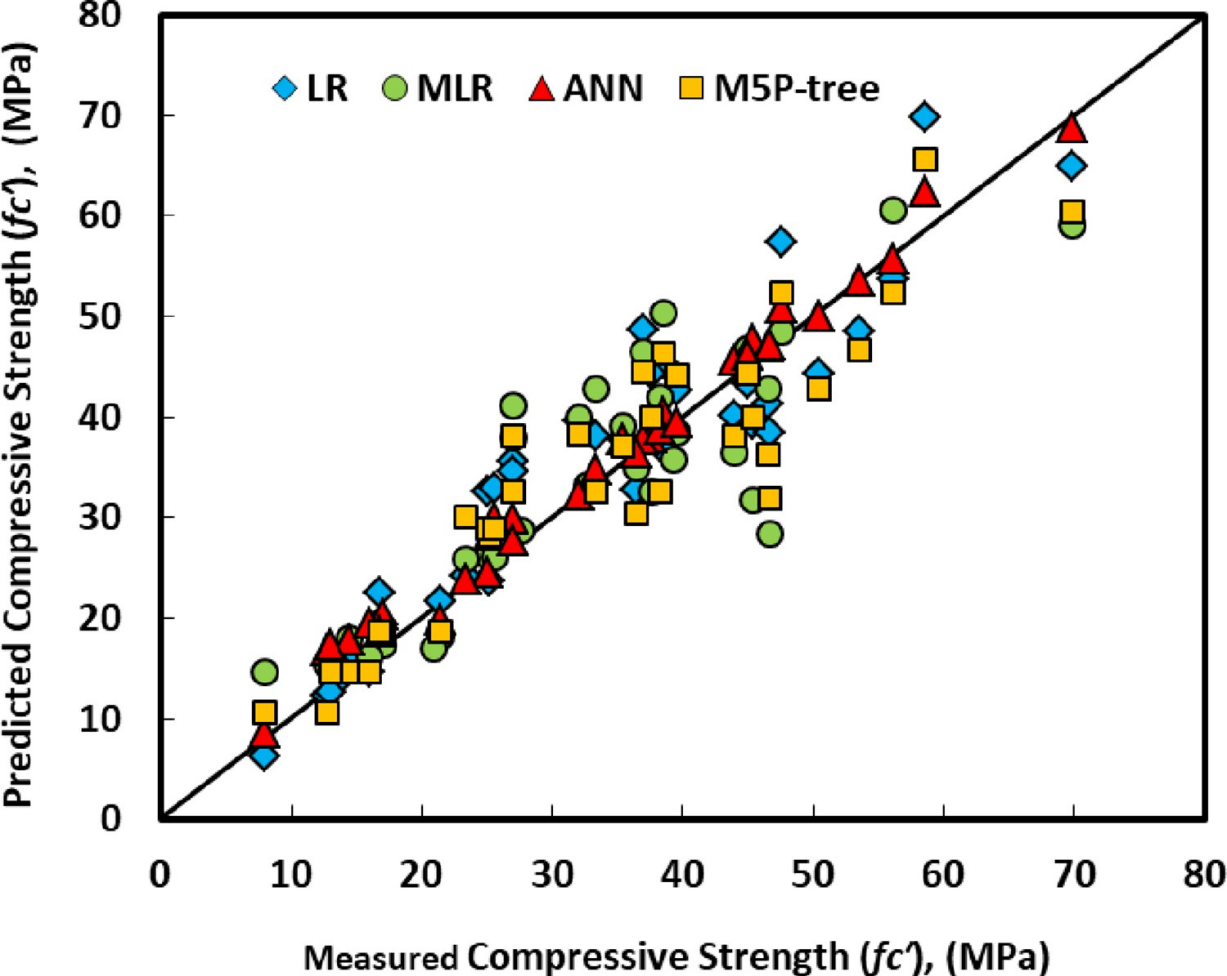
Model projections of compressive strength of GGBS/FA-GPC mixtures using testing data.

As mentioned previously, five different statistical tools, RMSE, MAE, SI, OBJ, and R2, were used to evaluate the efficiency of the developed models. The ANN model has higher R2 with lower RMSE and MAE values among the four different models compared to LR, MLR, and M5P models. Also, Fig 21 presents the comparison between model predictions of the compressive strength of GGBS/FA-GPC mixtures using testing data. Moreover, Fig 19 shows the residual error for all models using training, testing, and validating datasets. It can be noticed from both figures that the predicted and measured values of compressive strength are closer for the ANN model, which indicates the superior performance of the ANN model compared to other models.

The OBJ values for all proposed models are given in Fig 14. The value of OBJ for LR, MLR, ANN, and M5P is 4.685, 6.605, 1.929, and 4.278. The OBJ value of the ANN model is 142.8% less than the LR model, 242.4% lower than the MLR, and 121.7% lower than the M5P models. This also demonstrates that the ANN model is more efficient for predicting the compressive strength of GGBS/FA-GPC mixtures. Furthermore, the SI assessment parameter values for the proposed models in training, validating, and testing phases are presented in Fig 15. As shown in Fig 15, for all models and all phases (Training, testing, and validating), the SI values were between 0.1 and 0.2, indicating good performance for all models. However, similar to the other performance parameters the ANN model has lower SI values than other models. The ANN model has 88.7%, 174.6%, and 88.7% lower SI values than LR, MLR, and M5P models. This also illustrated that the ANN model is more efficient and performed better compared to LR, MLR, and M5P models for predicting the compressive strength of GGBS/FA-GPC.

## 7. Sensitivity of the models

A sensitivity comparison was carried out for the LR and MLR models in order to discover and analyze the most critical input parameter that impacts the compressive strength of GGBS/FA-GPC [57, 60]. In Excel, Solver created the training dataset for the models. Several alternative training data sets were employed in the sensitivity analysis. According to the obtained results (Table 3), the most important variables for the prediction of the compressive strength of GGBS/FA-GPC for the above stated two models are l/b, FA content, and M, and this is consistent with a number of studies [33, 35, 44, 66, 68, 69, 74, 85, 105–109].

**Table 3:** Sensitivity analysis using LR and MLR models.

		LR Model					MLR Model				
		R^2^	RMSE (MPa)	MAE (MPa)	OBJ	SI	R^2^	RMSE (MPa)	MAE (MPa)	OBJ	SI
**Removed Parameter**	**None**	0.902	4.655	3.604	2.960	0.134	0.806	6.621	5.171	4.504	0.193
	***l/b***	**0.876**	**5.235**	**3.968**	**3.344**	**0.150**	**0.802**	**6.686**	**5.225**	**4.506**	**0.192**
	***FA***	**0.875**	**5.253**	**3.972**	**3.354**	**0.151**	**0.764**	**7.297**	**5.481**	**4.938**	**0.209**
	***Si/Al***	0.896	5.843	4.914	3.867	0.172	0.799	7.875	6.602	5.323	0.231
	***GGBS***	0.898	4.748	3.664	3.021	0.136	0.778	7.082	5.465	4.811	0.203
	***Si/Ca***	0.898	4.766	3.725	3.051	0.137	0.806	6.625	5.171	4.454	0.190
	***F***	0.897	4.778	3.763	3.070	0.137	0.802	6.698	5.226	4.513	0.192
	***C***	0.896	4.793	3.765	3.077	0.138	0.800	6.722	5.248	4.534	0.193
	***SH***	0.897	4.785	3.771	3.076	0.138	0.775	7.134	5.406	4.817	0.204
	***SS***	0.893	4.877	3.732	3.101	0.140	0.776	7.113	5.380	4.796	0.204
	***SS/SH***	0.901	4.678	3.592	2.966	0.134	0.773	7.160	5.171	4.741	0.205
	***M***	**0.880**	**5.150**	**4.082**	**3.348**	**0.148**	**0.804**	**6.658**	**5.209**	**4.485**	**0.191**

## 8. Conclusions

Predicting of compressive strength of GGBS/FA-GPC by the reliable and accurate model can save time and cost. In this paper, linear regression (LR), multi-logistic regression (MLR), artificial neural network (ANN), and M5P-tree (M5P) models were used to propose predictively models for the compression strength of GGBS/FA-GPC. Based on the 220 collected datasets from previous research works and the simulation of the compressive strength of the GGBS/FA-GPC at the age of 28 days and ambient curing conditions, the following conclusion can be drawn:

1. All the used models LR, MLR, ANN, and M5P could be successfully used to develop predictive models for predicting the compressive strength of the GGBS/FA-GPC. The predicted compressive strengths matched well with the corresponding measured experimental compressive strengths of GGBS/FA-GPC.
2. The ANN model performs better than the other three models based on the statistical assessment and sensitivity analysis. The R^2^ values for this model are 0.988, 0.995, and 0.998 for the training, testing, and validating datasets, respectively. In addition, other sensitivity indicators for the training dataset for the ANN model are 2.478 MPa, 1.872 MPa, 1.929, and 0.071 for the RMSE, MAE, OBJ, and SI, respectively. So, the ANN model possesses a higher generality and predictive capability and is appropriate to practice in the preliminary design of GGBS/FA-GPC.
3. This study reported that the two-layer ANN model with 6 neurons in each layer is the suitable model combination for predicting GGBS/FA-GPC compressive strength.
4. The assessment and comparison of statistical parameters R^2^, RMSE, MAE, OBJ, and SI for all the training, testing, and validating datasets correctly validate the developed models’ accuracy.
5. The predicted compressive strengths with the ANN model were within +10% and -15% of the measured compression strength for the training datasets. However, this value was increased to ±20% for the other remaining models.
6. The obtained results show that the *l/b*, *FA* content, and *M* are the most significant variable parameters for predicting the compressive strength of GGBS/FA-GPC.
7. The proposed models could provide a detailed and practical foundation for increasing the re-use of waste GGBS and FA for construction practices instead of disposal in landfill sites.
8. The eco-efficient GGBS/FA-based geopolymer concrete studied here can participate in sustainable development because it is a cementless concrete and uses industrial or agro by-product ashes as a binder material; these mixture properties lead to a reduction of the carbon dioxide percent in the air, energy consumption, as well as waste disposal and the cost of the construction.
