# Expression of Concern: Soft computing models to predict the compressive strength of GGBS/FA- geopolymer concrete

**DOI:** 10.1371/journal.pone.0350324

**Published:** 2026-05-28

**Authors:** The PLOS One Editors

The *PLOS One* Editors issue this Expression of Concern because this article [1] was identified as one of a series of submissions for which we have concerns about the peer review process.

In addition, the articles cited as References 102 and 104 were retracted after [1] was published.

Readers are advised to interpret the article [1] with caution.
